# ERK and p38MAPK combine to improve survival in patients with BRAF mutant colorectal cancer

**DOI:** 10.1038/s41416-018-0174-y

**Published:** 2018-07-10

**Authors:** Antonia K. Roseweir, Elaine S. Halcrow, Sergey Chichilo, Arfon GMT Powell, Donald C. McMillan, Paul G. Horgan, Joanne Edwards

**Affiliations:** 1 Academic Unit of Surgery, School of Medicine-University of Glasgow, Royal Infirmary, Glasgow, G4 OSF UK; 2 Institute of Cancer Sciences, University of Glasgow, Wolfson Wohl Cancer Research Centre, Glasgow, G61 1QH UK; 3 Division of Cancer and Genetics, Cardiff University, Heath Park, Cardiff, CF14 4XN UK

**Keywords:** Colorectal cancer, Immunohistochemistry, Tumour biomarkers, Prognostic markers

## Abstract

**Background:**

In colorectal cancer (CRC), BRAF mutations influence tumour progression. In mismatch repair-deficient (dMMR) tumours, BRAF mutations are associated with a good prognosis, whereas in MMR-competent tumours, they are detrimental. The differential expression of the downstream MAPK pathway members, which are constitutively activated in BRAF mutant patients, may account for these differences.

**Methods:**

Phosphorylation of ERK, p38MAPK and JNK was assessed by immunohistochemistry, utilising CRC tissue microarrays. A discovery cohort (*n* = 187) and a validation cohort (*n* = 801) were analysed for associations with BRAF mutations, clinicopathological characteristics and cancer-specific survival (CSS).

**Results:**

In 801 CRC patients, nuclear ERK phosphorylation (HR 0.65 95% CI 0.48–0.88, *p* = 0.004) and the combined nuclear pERK/p-p38 score (HR 0.61 95% CI 0.45–0.82, *p* = 0.001) were independently associated with CSS, and were further associated with increased BRAF mutations (*p* = 0.003 and *p* = 0.002). When stratified for BRAF status, only MMR-competent patients harbouring the mutation and a strong combined nuclear pERK/p-p38 score (HR 0.49 95% CI 0.27–0.89, *p* = 0.016) demonstrated improved CSS. This improvement in CSS was specific to stage III CRC (HR 0.25 95% CI 0.10–0.64, *p* = 0.002).

**Conclusions:**

MMR-competent stage III tumours harbouring BRAF mutations have an improved prognosis when strong nuclear phosphorylation of both ERK and p38MAPK is present.

## Introduction

Colorectal cancer (CRC) is the second most common cause of cancer death in Europe.^1^ Although survival has improved, this is predominantly a result of better surgical technique and adjuvant/neo-adjuvant therapies. Despite this, 5-year survival remains poor, at 60%, across all stages of the disease.^2^ The present TNM-based staging of CRC is suboptimal, given the heterogeneity in survival among patients across the same stage of disease. There is an obvious clinical need to identify characteristics pertaining to both the tumour and the host, which may not only guide prognosis, but also novel adjuvant therapies.

BRAF V600E mutations are currently being investigated as a predictive biomarker for selecting patients for EGFR inhibitor treatment.^3^ BRAF mutations exhibit different associations with prognosis depending on the subset of CRC patients. In sporadic CRC, patients with mismatch repair (MMR)-deficient (dMMR) tumours harbouring BRAF mutations have an improved survival. Conversely, in MMR-competent (cMMR) patients, BRAF mutations convey a poor prognosis and their prognosis declines further in BRAF mutant metastatic disease.^4^ Furthermore, dMMR/BRAF mutant patients have a 5-year survival rate of 65% compared to 46% for cMMR/BRAF mutant patients. The reason for this difference in survival of BRAF mutant patients is still unclear, but may lie in the expression of downstream targets, such as extracellular regulated kinase (ERK).

ERK is part of the mitogen-activated protein kinase (MAPK) family along with two other members, p38MAPK and c-Jun-regulated kinase (JNK). All MAPKs are serine–threonine kinases activated by dual phosphorylation. The effects of MAPKs on patient survival in various cancers are varied,^5–8^ with some studies showing a survival advantage and some a detrimental survival effect. Most studies look at the three members in isolation, overlooking potential crosstalk between the pathways, which may explain the different associations with survival. Therefore, the aim of the present study was to assess the effect of phosphorylation of ERK, p38MAPK and JNK alone and in combination on cancer-specific survival (CSS) in a discovery and validation cohorts of CRC patients. The study also assessed the associations between the MAPK pathway, BRAF mutations, MMR status and clinicopathological factors.

## Methods

### Patients

The discovery cohort patients (*n* = 272) were identified from retrospectively retrieved routine CRC resections performed within the Glasgow Royal Infirmary between 1997 and 2007. This cohort was extended to the validation cohort (*n* = 1030) with the addition of retrospectively identified CRC resections performed in the Western Infirmary and Stobhill Hospital, Glasgow in the same time period. Patients who had undergone a potentially curative resection for stage I–III CRC and were included within previously constructed tissue microarrays (TMAs) were studied. Resections were considered curative based on pre-operative computed tomography and intra-operative findings. Patients who had died within 30 days of surgery were excluded. Ethical approval was obtained from the West of Scotland Research Ethics Committee.

### Clinicopathological characteristics

Tumours were staged using the fifth edition of the AJCC/UICC-TNM staging system.^9^ Tumour differentiation was graded in accordance with Royal College of Pathologists.^10^ The presence of venous invasion was assessed using Elastica staining. Differentiation, margin involvement, peritoneal involvement and necrosis were taken from pathology reports issued following resection. Data on Ki67 were already available for both the cohorts using a threshold of 50%. MMR status was assessed as previously described.^11^ Patients were followed up for at least 5 years, and date and cause of death were crosschecked with electronic case records. CSS was measured from the date of surgery to the date of death from CRC.

### Assessment of inflammatory responses

Stromal infiltration was assessed using tumour stroma percentage (TSP) as previously described.^12^ The local inflammatory cell infiltrate was assessed using the Klintrup–Makinen (KM) grade.^13^ The Glasgow microenvironment score (GMS) was calculated as previously described.^14^ Tumour-infiltrating lymphocyte (TIL) counts were established from pathology reports issued following resection. For systemic inflammation, serum C-reactive protein (CRP) and albumin were recorded prospectively and measured within 30 days prior to surgery. The pre-operative systemic inflammatory response was defined using the modified Glasgow prognostic score (mGPS).^15^

### Immunohistochemistry

BRAF V600E, phosphorylated ERK1/2 (pERK), phosphorylated p38MAPK (p-p38) and phosphorylated JNK (pJNK) were assessed via immunohistochemistry (Figure S1) in the discovery (*n* = 272) and validation TMAs (*n* = 758). Antibody validation was performed with a single band on a western blot and EGF or UV-stimulated cell pellet +/- inhibitors. For BRAF V600E, the antibody was validated using BRAF WT and BRAF V600E mouse colon tissue (Figure S2).

Sections were dewaxed in histoclear and then rehydrated using graded alcohols. Antigen retrieval for pERK was performed in citrate buffer at 96 °C for 20 min or for BRAF V600E, p-p38 and pJNK using Tris EDTA buffer pH 8 under pressure for 5 min. Endogenous peroxidase activity was blocked using 3% hydrogen peroxidase. 10% casein (BRAF V600E), 1.5% horse serum (pERK) or 5% horse serum (p-p38/pJNK) was applied as a blocking solution. TMA sections were incubated overnight at 4 °C with primary BRAF V600E (1:200, clone VE1, Spring Biosciences #E1929), pJNK (Thr183/Tyr185, 1:50, cell signalling #4668) or p-p38 (Thr180/Tyr182, 1:100, cell signalling #4511) antibody. Primary pERK (Thr202/Tyr204, 1:200, cell signalling #9101) antibody was incubated for 6 h at room temperature. Envision (Dako) was used as a secondary antibody before DAB substrate was added for colour development. Slides were counterstained with haematoxylin and blued with Scott’s tap water before being dehydrated through a series of graded alcohols and histoclear. Coverslips were applied using distyrene, plasticizer, xylene (DPX).

### Scoring

Stained TMA sections were scanned using a Hamamatsu NanoZoomer (Welwyn Garden City, Hertfordshire, UK) at ×20 magnification and visualised on Slidepath Digital Image Hub (Leica Biosystems, Milton Keynes, UK). Assessments of BRAF V600E, ERK1/2, JNK and p38MAPK phosphorylation were performed by a single examiner blinded to clinical data at ×20 magnification (total magnification ×400) using the weighted histoscore. The weighted histoscore was calculated as follows: 0×% not stained + 1×% weakly stained + 2×% moderately stained + 3×% strongly stained. This gave a range of scores from 0 to 300, with cytoplasmic and nuclear staining scored separately. A total of 10% of tumours were co-scored by a co-investigator, and the interclass correlation coefficient calculated to be <0.7 for all proteins.

### Statistical analysis

Histograms were assessed for each protein, and BRAF V600E and pERK histograms determined that negative and positive expressions were the appropriate thresholds. Receiver operator characteristic (ROC) curves were employed to identify the optimal threshold for low/high expression of p-p38 and pJNK in the discovery cohort and validated using the validation cohort; the following thresholds were identified for each protein: 40 for nuclear p-p38, 10 for cytoplasmic p-p38, 70 for nuclear pJNK and 145 for cytoplasmic pJNK.

SPSS (version 22) was used for statistical analysis. By using a two-sided α = 0.05 analysis and assuming a hazard ratio (HR) of 0.65 and a both high prevalence of 40% for the combined nuclear score, a sample size of >178 patients gave >90% power to detect a survival difference between the both low, or one high and both high groups. Pearson’s *χ*^2^ test assessed the associations between MAPK members, BRAF mutations and clinicopathological features. Kaplan–Meier curves and log-rank analysis compared CSS. HRs and confidence intervals (CI) were calculated from univariate Cox regression survival analysis. Multivariate Cox regression survival analysis using a backward conditional elimination model and a significance threshold of *p* < 0.05 was performed to identify independent prognostic biomarkers. The study is reported in line with the REMARK guidelines,^16^ and significance was set as *p* < 0.05.

## Results

For the discovery cohort, a total of 187 patients were studied, and they underwent a potentially curative resection for stage I–III CRC and had a valid score for pERK, p-p38 and pJNK. The patient characteristics for the discovery cohort are shown in Table S1. In brief, 64% were 65 years or older and 53% were male. Five percent had stage I disease, 49% had stage II disease and 46% had stage III disease. Thirty-eight percent had right-sided colon cancer, 30% had left-sided colon cancer and 32% had rectal cancer. MMR deficiency was identified in 10% of  patients and 20% had BRAF V600E mutations. Thrity-one percent received adjuvant chemotherapy. The median follow-up for patients was 11.6 years (range 7.3–16.0 years), with 65 cancer deaths and 45 non-cancer deaths.

The association between MAPK phosphorylation and CSS within the cytoplasm or nucleus was investigated in Table 1. p-p38 and pJNK were not associated with CSS at either cellular location. However, phosphorylation of ERK significantly improved CSS within the nucleus (HR 0.61 95% CI 0.38–1.00, *p* = 0.048). To assess if analysing the MAPK family in combination would provide any additional power, combined prognostic scores were established as follows: combined pERK/p-p38 score, combined pERK/pJNK score and combined p-p38/pJNK score. For all combined scores, patients were split into two groups; patients with weak activation of both proteins or strong activation of one protein were termed as both weak or one strong, and patients with strong activation of both proteins were termed as both strong. Only the combined nuclear pERK/p-p38 score provided some additional power to improve CSS (HR 0.56 95% CI 0.33–0.95, *p* = 0.030, Table 1). Next, to assess if this increased power is relevant, this combined pERK/p-p38 score and pERK alone were taken forward into the validation cohort.

**Table 1:** Phosphorylation of MAPK and cancer-specific survival in discovery cohort patients undergoing potentially curative resection of colorectal cancer (*n* = 187)

	Nuclear			Cytoplasmic		
	*N (*%)	10 yr CSS (SE)	*p*	*N (%)*	10 yr CSS (SE)	*p*
*pERK1/2*						
Weak activation	71 (38)	55 (7)	0.048*	69 (37)	55 (7)	0.058
Strong activation	116 (62)	69 (5)		118 (63)	69 (5)	
*p-p38MAPK*						
Weak activation	67 (36)	58 (6)	0.237	143 (76)	64 (4)	0.985
Strong activation	120 (64)	67 (5)		44 (24)	61 (8)	
*pJNK*						
Weak activation	86 (46)	62 (6)	0.644	65 (35)	75 (6)	0.055
Strong activation	101 (54)	65 (5)		122 (65)	58 (5)	
*Combined pERK/p-p38 score*						
Both weak or one strong	110 (59)	57 (5)	0.03*	161 (86)	62 (4)	0.214
Both strong	77 (41)	73 (5)		26 (14)	75 (9)	
*Combined pERK/pJNK score*						
Both weak or one strong	129 (69)	61 (5)	0.217	112 (55)	66 (5)	0.67
Both strong	58 (31)	70 (6)		75 (45)	61 (6)	
*Combined p-p38/pJNK score*						
Both weak or one strong	121 (65)	60 (5)	0.245	156 (83)	64 (4)	0.724
Both strong	66 (35)	69 (6)		31 (17)	64 (9)	

*Significant difference *p* < 0.05.

To validate theses findings, the cohort was extended to an 801 CRC patient validation cohort. CRC patients with a valid score for pERK and p-p38 were included. The characteristics of this cohort are shown in Table S1. In Brief, 68% were 65 years or older and 53% were male. Fourteen percent had stage I disease, 48% had stage II disease, and 38% had stage III disease. Forty-one percent had right-sided colon cancer, 34% had left-sided colon cancer and 24% had rectal cancer. MMR deficiency was identified in 16% of  patients and 21% had BRAF V600E mutations. The median follow-up for patients was 12.0 years (range 7.3–16.0 years), with 235 cancer deaths and 258 non-cancer deaths. As this data was similar to that of the discovery cohort, it was deemed appropriate to validate the findings.

The validation cohort was assessed for associations with CSS (Table 2); p-p38 did not associate with CSS at either cellular location. However, pERK was associated with improved CSS within both the nucleus (HR 0.76 95% CI 0.59–0.99, *p* = 0.037) and cytoplasm (HR 0.77 95% CI 0.60–0.99, *p* = 0.047). When the combined pERK/p-p38 score was assessed, associations with improved CSS were strengthened for both nuclear (HR 0.69 95% CI 0.53–0.90, *p* = 0.005) and cytoplasmic localisation (HR 0.68 95% CI 0.51–0.92, *p* = 0.010).

**Table 2:** Phosphorylation of MAPK and cancer-specific survival in validation cohort patients undergoing potentially curative resection of colorectal cancer (*n* = 801)

	Nuclear			Cytoplasmic		
	*N* (%)	10 yr CSS (SE)	*P*	*N* (%)	10 yr CSS (SE)	*P*
*pERK1/2*						
Weak activation	327 (41)	64 (3)	0.037*	379 (47)	65 (3)	0.047*
Strong activation	474 (59)	72 (2)		422 (53)	72 (2)	
*p-p38MAPK*						
Weak activation	251 (31)	65 (3)	0.236	380 (47)	66 (3)	0.268
Strong activation	550 (69)	70 (2)		421 (53)	71 (2)	
***C*** *ombined pERK/p-p38 score*						
Both weak or one strong	427 (53)	64 (2)	0.005*	536 (67)	65 (2)	0.01*
Both strong	374 (47)	74 (2)		265 (33)	76 (3)	

*Significant difference *p* < 0.05

pERK and the combined pERK/p-p38 score were then assessed for associations with clinicopathological factors as shown in Table 3. Patients with phosphorylation of nuclear ERK were more likely to have a BRAF V600E mutation (*p* = 0.003) and be MMR competent (*p* = 0.010), and have lower CRP levels (*p* = 0.030). Similarly, patients with phosphorylation of cytoplasmic ERK were more likely to have a BRAF V600E mutation (*p* = 0.009) and be MMR competent (*p* = 0.032). For the combined nuclear pERK/p-p38 score, patients with a both strong score were associated with increased BRAF V600E mutations (*p* = 0.002). Whereas a both strong score for the combined cytoplasmic pERK/p-p38 score was associated with older age (*p* = 0.039). No associations with other clinicopathological characteristics or local inflammation were seen for any MAPK members.

**Table 3:** Clinicopathological characteristics of validation patients undergoing potentially curative resection of colorectal cancer and cancer-specific survival (*n* = 801)

	Nuclear pERK1/2			Cytoplasmic pERK1/2			Combined nuclear pERK/p38 score			Combined cytoplasmic pERK/p38 score		
	Weak (*n* = 327)	Strong (*n* = 474)	*p*	Weak (*n* = 379)	Strong (*n* = 422)	*p*	Bothweak/one strong (*n* = 427)	Both strong (*n* = 374)	*p*	Both weak/one strong (*n* = 536)	Both strong (*n* = 265)	*p*
*BRAF status*												
Wild type	265 (84)	352 (75)	0.003*	306 (83)	331 (75)	0.009*	344 (83)	273 (74)	0.002*	416 (80)	201 (76)	0.228
Mutant	50 (16)	115 (25)		63 (17)	102 (25)		70 (17)	95 (26)		103 (20)	62 (24)	
*Mismatch repair status*												
Competent	529 (80)	405 (86)	0.01*	302 (81)	362 (86)	0.032*	343 (81)	321 (86)	0.065	439 (83)	225 (85)	0.526
Deficient	66 (20)	63 (14)		72 (19)	57 (14)		78 (19)	51 (14)		89 (17)	40 (15)	
*Age*												
<65	108 (33)	148 (31)	0.591	125 (33)	131 (31)	0.557	140 (33)	116 (31)	0.592	184 (34)	72 (27)	0.039*
>65	219 (67)	326 (69)		254 (67)	291 (69)		287 (67)	258 (69)		352 (66)	193 (73)	
*Serum CRP*												
Normal	137 (51)	222 (59)	0.03*	165 (55)	194 (57)	0.595	191 (53)	168 (59)	0.172	256 (56)	103 (54)	0.547
High	133 (49)	152 (41)		137 (45)	148 (43)		167 (47)	118 (41)		197 (44)	88 (46)	
*mGPS*												
0	138 (51)	224 (59)	0.069	168 (55)	194 (57)	0.776	192 (53)	170 (59)	0.243	259 (57)	103 (54)	0.192
1	89 (33)	97 (26)		95 (31)	91 (26)		112 (31)	74 (26)		135 (29)	51 (27)	
2	45 (16)	55 (15)		42 (14)	58 (17)		57 (16)	43 (15)		63 (14)	37 (19)	
*Tumour-infiltrating lymphocytes*												
Absent	242 (76)	368 (79)	0.297	293 (79)	317 (77)	0.527	313 (76)	297 (81)	0.061	398 (76)	212 (82)	0.078
Present	75 (24)	95 (21)		77 (21)	93 (23)		101 (24)	69 (19)		123 (24)	47 (18)	
*Klintrup–Makinen grade*												
Weak	217 (68)	317 (68)	0.966	257 (69)	277 (67)	0.514	276 (66)	258 (70)	0.31	362 (69)	172 (66)	0.451
Strong	102 (32)	150 (32)		115 (31)	137 (33)		140 (34)	112 (30)		164 (31)	88 (34)	
*TNM stage*												
1	45 (14)	65 (14)	0.567	51 (14)	59 (14)	0.152	61 (14)	49 (13)	0.996	68 (13)	42 (16)	0.052
2	152 (46)	234 (49)		171 (45)	215 (51)		201 (47)	185 (50)		252 (47)	134 (51)	
3	130 (40)	175 (37)		157 (41)	148 (35)		165 (39)	140 (37)		216 (40)	89 (33)	
*Venous invasion*												
Absent	218 (67)	318 (67)	0.901	254 (67)	282 (67)	0.954	281 (66)	255 (68)	0.476	353 (66)	183 (69)	0.364
Present	109 (33)	156 (33)		125 (33)	140 (33)		146 (34)	119 (32)		183 (34)	82 931)	
*Proliferation Index*												
Low	146 (45)	213 (45)	0.943	169 (45)	190 (46)	0.835	197 (47)	162 (44)	0.412	235 (44)	124 (47)	0.483
High	178 (55)	259 (55)		208 (55)	227 (54)		226 (53)	209 (56)		295 (56)	140 (53)	

*Significant difference *p* < 0.05

**Fig. 1:**
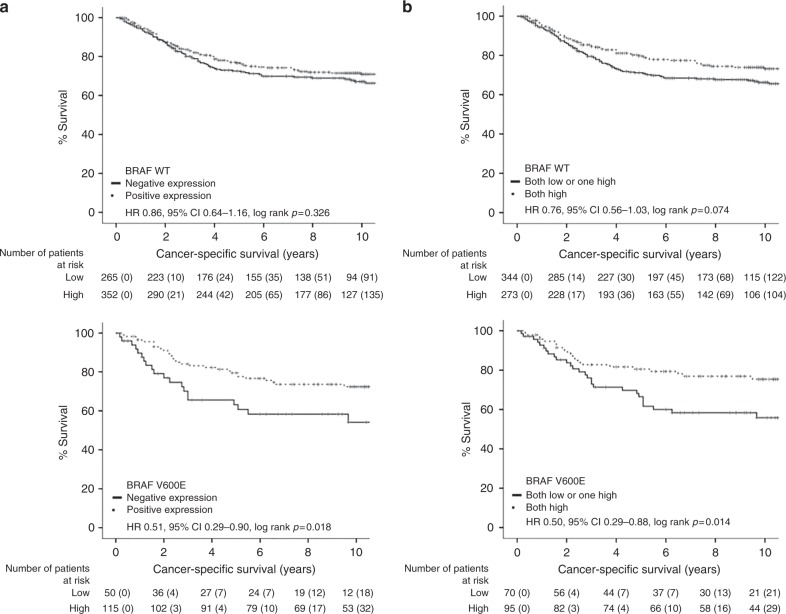
Combined nuclear pERK/p-p38 score stratifies CSS in BRAF mutant CRC patients (*n* = 782). Kaplan–Meier curves showing associations between (**a**) pERK or (**b**) the combined nuclear pERK/p-p38 score and CSS in BRAF WT and BRAF mutant patients with CRC

**Fig. 2:**
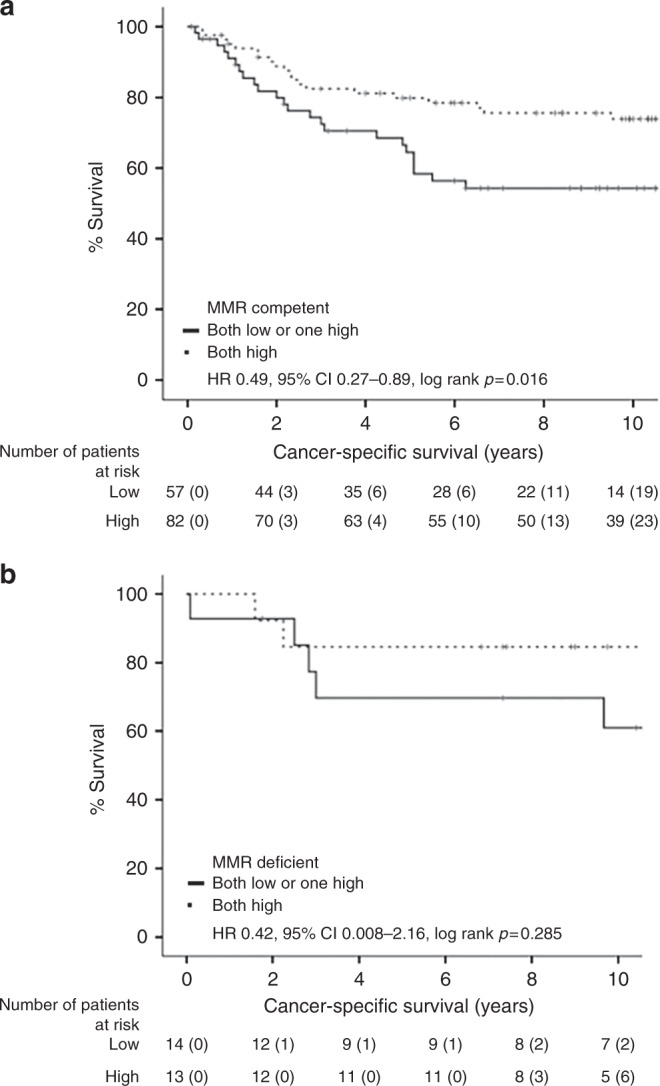
Combined nuclear pERK/p-p38 score differentially stratifies BRAF mutant CRC patient survival in MMR-competent and MMR-deficient patients (*n* = 165). Kaplan–Meier curves showing association between the combined nuclear pERK/p-p38 score and CSS in BRAF mutant patients with (**a**) MMR-competent or (**b**) MMR-deficient CRC

As the combined nuclear pERK/p-p38 score is associated with increased BRAF mutations, as well as pERK alone, firstly, associations between nuclear p-p38 and BRAF status were assessed as they are normally within divergent MAPK pathways. High nuclear p-p38 did associate with increase BRAF mutations (*p* = 0.039, Table S2), suggesting that they do interact. Next, patients were stratified into BRAF wild type or BRAF mutant, and effects on the patient's CSS was assessed (Fig. 1). For phosphorylation of nuclear ERK, CSS was only improved in patients with BRAF mutations (HR 0.51 95% CI 0.29–0.90, *p* = 0.018, Fig. 1a). This effect on CSS was slightly potentiated for the combined nuclear pERK/p-p38 score (HR 0.50 95% CI 0.29–0.88, *p* = 0.014, Fig. 2b). To further assess BRAF mutant patients, the combined nuclear pERK/p-p38 score was assessed for effects pertaining to MMR status (Fig. 2). In cMMR patients, a both strong combined nuclear pERK/p-p38 score improved CSS (HR 0.49 95% CI 0.27–0.89, *p* = 0.016). Whereas in MMR-deficient patients, a both strong combined pERK/p-p38 score did not significantly improve patient survival (HR 0.45 95% CI 0.08–2.16, *p* = 0.285).

To further investigate the utility of the combined nuclear pERK/p-p38 score within BRAF mutant patients, patients were further stratified for TNM stage. For cMMR patients (Figure S3), only BRAF mutant patients with stage III CRC had improved survival with a both strong score (HR 0.25 95% CI 0.10–0.64, *p* = 0.002, Figure S3C). No survival advantage was seen for stage I (HR 1.84 95% CI 0.19–17.94, *p* = 0.593, Figure S3A) or stage II patients (HR 0.97 95% CI 0.36–2.63, *p* = 0.952, Figure S3B). No effect of stage was seen in MMR-deficient patients.

pERK and the combined pERK/p-p38 score were then taken forwards into multivariate analysis with common clinicopathological factors (Table 4). Under multivariate analysis for all patients (*n* = 606), TNM stage (*p* < 0.001), venous invasion (*p* = 0.006), margin involvement (*p* = 0.032), peritoneal involvement (*p* < 0.001), TSP (*p* = 0.030), KM grade (*p* = 0.030), TILs (*p* = 0.002), mGPS (*p* < 0.001), nuclear pERK (*p* = 0.004) and the combined nuclear pERK/p-p38 score (*p* = 0.001) were independent prognostic factors for CSS. When stratified for cMMR BRAF mutant patients (*n* = 136), venous invasion (*p* = 0.003), margin involvement (*p* = 0.009), mGPS (*p* < 0.001) and nuclear pERK (*p* = 0.042) remained independently prognostic. When further stratified for stage III patients (*p* = 53), margin involvement (*p* = 0.029), mGPS (*p* = 0.001) and nuclear pERK (*p* < 0.001) remained independent for CSS.

**Table 4:** Multivariate analysis of MAPK phosphorylation, clinicopathological characteristics and cancer-specific survival in patients with colorectal cancer (*n* = 606)

	Multivariate HR (95% CI)	*p*
*All patients* *(n* = *606)*		
TNM stage (I/II/III)	1.87 (1.44–2.45)	<0.001*
Differentiation (moderate or well/poor)	1.10 (0.69–1.75)	0.703
Venous invasion (absent/present)	1.53 (1.13–2.07)	0.006*
Margin Invovlement (no/yes)	1.69 (1.05–2.73)	0.032*
Peritoneal invovlement (no/yes)	1.79 (1.31–2.45)	<0.001*
Proliferation index (low/high)	0.81 (0.60–1.10)	0.178
Tumour stroma percentage (low/high)	1.43 (1.04–1.99)	0.030*
Klintrup–Makinen grade (weak/strong)	0.64 (0.48–0.88)	0.030*
TILs (absent/present)	0.48 (0.30–0.77)	0.002*
mGPS (0/1/2)	1.57 (1.30–1.91)	<0.001*
Nuclear pERK (low/high)	0.65 (0.48–0.88)	0.004*
Combined nuclear pERK/p-p38 score (both low or one high/both high)	0.61 (0.45–0.82)	0.001*
*MMR-competent BRAF mutant patients* *(n* *=* *136)*		
TNM stage (I/II/III)	1.32 (0.74–2.38)	0.351
Venous invasion (absent/present)	2.58 (1.37–4.86)	0.003*
Margin Invovlement (no/yes)	4.45 (1.45–13.70)	0.009*
Peritoneal invovlement (no/yes)	0.95 (0.48–1.89)	0.887
Klintrup–Makinen grade (weak/strong)	0.72 (0.31–1.65)	0.436
TILs (absent/present)	0.42 (0.17–1.02)	0.054
mGPS (0/1/2)	2.66 (1.78–3.98)	<0.001*
Nuclear pERK (low/high)	0.52 (0.28–0.98)	0.042*
Combined nuclear pERK/p-p38 score (both low or one high/both high)	0.58 (0.31–1.06)	0.077
*Stage III MMR-competent BRAF mutant patients (n* *=* *53)*		
Venous invasion (absent/present)	1.17 (0.43–3.15)	0.762
Margin Invovlement (no/yes)	6.59 (1.21–35.87)	0.029*
Klintrup–Makinen grade (weak/strong)	0.19 (0.02–1.44)	0.107
TILs (absent/present)	0.21 (0.03–1.69)	0.143
mGPS (0/1/2)	3.01 (1.58–5.73)	0.001*
Nuclear pERK (low/high)	0.16 (0.06–0.44)	<0.001*
Combined nuclear pERK/p-p38 score (both low or one high/both high)	0.34 (0.10–1.19)	0.091

*Significant difference

## Discussion

The results of the present study suggest that patients with CRC need strong nuclear phosphorylation of both ERK and p38MAPK for a good prognosis. The data show that this survival improvement is enhanced in cMMR patients with stage III CRC harbouring BRAF mutations. However, patients with strong activation of only one protein have a poorer survival outcome, suggesting that these patients may benefit from ERK or p38MAPK activation.

ERK has long been associated with malignant transformation in various cancers including CRC, with upstream KRAS/BRAF harbouring driver mutations.^6,8,17^ Therefore, it is interesting that this improvement in patient survival is specific to BRAF- mutant tumours, with strong activation of ERK. Previous studies have mainly associated phosphorylation of ERK with reduced survival in patients with CRC,^18,19^ however, they assessed ERK in isolation, and therefore, the differences in the activation of p38MAPK between the cohorts may account for the difference in survival effects compared to the present study. p38MAPK is thought to suppress cell proliferation in normal cells, but can promote proliferation in certain cancer cells, and this has been linked to activation levels.^20^ This observation is similar to ERK, which has also been shown to adapt its proliferative effects depending on activation levels, with strong activation of ERK causing cell cycle arrest and decreased proliferation.^5^ Furthermore, p38MAPK has also been shown to suppress ERK activity, which may be important in BRAF mutant tumours where ERK is hyperactivated.^21^ In the current study, patients with BRAF mutant tumours have a threefold increase in nuclear ERK phosphorylation compared to BRAF WT tumours (data not shown), supporting the hypothesis that ERK is hyperactivated in BRAF mutant patients. Although p38MAPKs is thought to be a poor prognostic factor in CRC that promotes cancer cell survival,^22^ most research to date has been restricted to cell lines and mouse models.^23,24^ However, the present tissue data suggest that high levels of phosphorylated p38MAPK promote proliferation potentially by inhibiting ERK activation. Therefore, in patients with high phosphorylation of both nuclear p38MAPK and ERK, p38MAPK may dampen the anti-proliferative effects of ERK to maintain cell proliferation. This is in line with previous literature that suggests proliferation measured by Ki67 is a good prognostic factor in patients with CRC.^25,26^ However, if only ERK phosphorylation is high, then the hyperactivation in BRAF mutant tumours will be uncontrolled and start to suppress the cell cycle, leading to decreased proliferation and reduced patient survival.

This was observed when assessing the combined nuclear ERK/p38 score in BRAF mutant patients. In BRAF mutant patients with strong activation of both ERK and p38MAPK, survival is significantly improved. It is interesting to note that in 115 patients with strong activation of ERK, 95 patients also had strong activation of p38. This suggests that dual activation is common in BRAF mutant tumours and accounts for why only a slight increase in power is seen between nuclear pERK and the combined pERK/p-p38 score (Fig. 1). This dual activation protects the patient against the hyperactivation of ERK, allowing the tumour to continue to proliferate, which has been previously shown to convey a good prognosis to patients with CRC.^25^ However, when only one MAPK is highly active or both are weak, survival is decreased, which suggests that for a protective influence to ensue, high activation levels of both members are required. When only one member is activated, both proliferation and survival rates are lowered to a similar level to that observed for patients that have low activation of both. This suggests that proliferation needs to be driven by both members for improved patient prognosis. This effect is not seen in BRAF WT patients, suggesting that only when ERK is hyperactivated, can it affect the cell cycle inhibiting proliferation, which leads to a worse prognosis for these patients. In BRAF WT patients, the levels of ERK activation never reach the threshold to affect the cell cycle, so the reduction in proliferation and survival is never produced.

As BRAF mutations are commonly associated with MMR-deficient patients,^4^ we next stratified BRAF mutant patient by MMR status. In patients with cMMR CRC, similar results were seen with a both strong nuclear ERK/p38 score conveying a survival advantage to the patient, suggesting that this score would be a useful prognostic marker in these patients. To confirm the utility of this prognostic marker across all disease stages of CRC, we next stratified cMMR BRAF mutant patients by stages, and found that this survival advantage with the nuclear ERK/p38 score was potentiated in patients with stage III CRC. Patients with a both weak/one strong score had significantly poorer survival rates. In contrast, the survival difference previously observed was lost in stage I/II patients. These finding suggest that BRAF mutational analysis should be extended beyond it’s current clinical application in metastatic disease to the adjuvant setting to further aid clinicians with patient prognosis.

In conclusion, this is the first study to show a combined survival advantage of ERK and p38MAPK in cMMR BRAF mutant patients with stage III CRC; confirmation in an independent cohort is needed. One limitation of this study is that it does not cover metastatic disease and therefore further analysis of this combined score in BRAF mutant metastatic patients is also warranted. Overall, these results suggest that patients within the adjuvant setting with cMMR Stage III CRC should not only be routinely tested for BRAF mutations, but should also be further tested for phosphorylation of ERK and p38MAPK to fully stratify their prognosis. Furthermore, strong expression of only one of these proteins (ERK or p38MAPK) could be used as a predictive biomarker for clinical trials in BRAF mutant cMMR CRC patients, establishing the benefit of treatment with an agonist to the other member (ERK for p38MAPK agonist and p38MAPK for ERK agonist). This approach would assess if this combined MAPK score truly has a prognostic benefit and would help move towards a precision medicine approach for patients with CRC.

## Electronic supplementary material

Supplementary Data

## Availability of data and material

All clinical information is held within a database available from the NHS GGC Safehaven, and tissue microarrays are available from the NHS Research Scotland GGC Biorepository.
