# Supplementary material for: ERK and p38MAPK combine to improve survival in patients with BRAF mutant colorectal cancer
Source: Br J Cancer. 2018 Jul 10;119(3):323–9. doi: 10.1038/s41416-018-0174-y (PMC6070918; doi:10.1038/s41416-018-0174-y)
Supplement: Supplementary file 1 — Supplementary Data [file 41416_2018_174_MOESM1_ESM.docx]

**Supplementary Methods**

**Cell culture**

HT29 cells were maintained in McCoy’s 5A (Modified) Medium (ThermoFisher, UK) with 10% FBS, 1% GlutaMAX and 1% streptomycin/penicillin. T84 culture was maintained in a 1:1 mixture of Ham’s F12 medium and DMEM (ThermoFisher, UK) with 5% FBS, 1% GlutaMAX and 1% streptomycin/penicillin. All incubations were at 37 °C with 5% CO2.

**Preparation of Cellular Extraction and Immunoblotting.**

Cells were seeded at 1x10^5^ cells/well density in 6 well plates. Upon confluency, cells were serum starved overnight then treated with MAPK inhibitors; 20µM PD98095 (ERK1/2), 1µM SP600125 (JNK), and 1µM SB202190 (p38MAPK) for 24 hours. Cells were lysed using Nonidet P-40 (NP-40) buffer (ThermoFisher, UK) for 5 minutes on ice then scraped down. Lysates were centrifugation at 13,000 rpm at 4°C for 15 minutes then supernatant was collected and stored at 4°C until needed.

Samples were denatured at 100 °C and separated by SDS-PAGE at 120V for 90 minutes. Proteins were transferred to PVDF membrane (Millipore, UK) at 200mA for 60 minutes. The membrane was blocked using 5% Bovine Serum Albumin (BSA) for 1 hour. pERK1/2, pJNK, and p-p38 anti-rabbit primary anti-human antibodies (1:500) were incubated at 4°C overnight (Cell Signalling). HRP conjugated polyclonal anti-rabbit IgG secondary antibody diluted at 1:5000 was added for 1 hour. Visualisation was performed using enzyme-linked chemiluminescense (ECL) and imaged using the G:BOX Chemi-XX6 (SYNGENE UK). Equal protein loading was confirmed by anti b-tubulin (Abcam UK).

**Cell Pellet stimulation and Preparation**

Cells were plated at 3x10^5^ cells/well in 6 well plates. Upon confluency, cells were pre-treated with MAPK inhibitors; 20µM PD98095 (ERK1/2), 1µM SP600125 (JNK), and 1µM SB202190 (p38MAPK) for 1 hours. Then either EGF stimulation for 30 minutes (ERK) or UV stress for 30 minutes (JNK/p38MAPK). Cells were then trypsinized and pelleted at 1200rpm for 5 mins. Pellets were washed with PBS then centrifuged at 2500rpm for 3 minutes. Pellets were fixed in formalin for 15 minutes, then spun down at 2500rpm for 3 mins followed by washing in PBS. After air drying, pellets were coated in agarose overnight at 4C. Pellets were then dehydrated in alcohol, penetrated with wax and then placed in wax blocks. Sections were then cut a 4um and baked on glass slides overnight at 50C. Then immunohistochemistry was performed as for the TMA sections.

**Table S1. Patient characteristics for cohorts**

|  | **Discovery cohort**  **n=187 (%)** | **Validation Cohort**  **n=801 (%)** |
| --- | --- | --- |
| **Age**  **<65**  **>65** | 68 (36)  119 (64) | 256 (32)  545 (68) |
| **Sex**  **Female**  **Male** | 87 (47)  100 (53) | 380 (47)  421 (53) |
| **TNM-stage**  **I**  **II**  **III** | 9 (5)  81 (49)  87 (46) | 110 (14)  386 (48)  305 (38) |
| **T-stage**  **1**  **2**  **3**  **4** | 5 (3)  12 (6)  118 (63)  52 (28) | 33 (4)  98 (12)  441 (55)  229 (29) |
| **N-stage**  **0**  **1**  **2** | 100 (53)  67 (36)  20 (11) | 499 (62)  213 (27)  89 (11) |
| **Tumour Location**  **Right-sided colon**  **Left-sided colon**  **Rectal**  **Unknown** | 71 (38)  56 (30)  60 (32)  0 (0) | 326 (41)  276 (34)  194 (24)  5 (1) |
| **Adjuvant Therapy**  **No**  **Yes** | 129 (69)  58 (31) | -  - |
| **Differentiation**  **Moderate/well**  **Poor** | 167 (89)  20 (11) | 725 (90)  76 (10) |
| **Venous Invasion**  **Absent**  **Present** | 116 (62)  71 (38) | 536 (67)  265 (33) |
| **MMR Status**  **Competent**  **Deficient**  **Unknown** | 163 (87)  19 (10)  5 (3) | 664 (83)  129 (16)  8 (1) |
| **BRAF V600E**  **WT**  **mutant**  **Unknown** | 138 (74)  38 (20)  11 (6) | 617 (77)  165 (21)  19 (2) |
| **Survival**  **Alive**  **Cancer Death**  **Non-cancer Death** | 77 (41)  65 (39)  45 (24) | 308 (39)  235 (29)  258 (32) |

**Table S2. P38MAPK associates with increased BRAF V600E mutations**

|  | **Nuclear p38MAPK** | | |
| --- | --- | --- | --- |
|  | **Weak activation**  **(n=246)** | **Strong activation**  **(n=543)** | **P-value** |
| BRAF status  WT  V600E | 205 (83)  41 (17) | 418 (77)  125 (23) | 0.039 |

**Figure S1. Immunohistochemical Staining (n=231).** Representative images of negative, low moderate and high immunohistochemical staining for each of the antibodies taken at x400 magnification.

**Figure S2. Validation of IHC antibodies** (A) Western blot images showing a single band for each antibody in unstimulated HT29 (BRAF V600E) or T84 (BRAF WT) cell lines. Total ERK was used as a loading control. (B-d) Cell pellets for HT29 and T84 cells treated with (B) EGF +/- PD98059 inhibitor and stained wth pERK antibody (C) UV stress +/- SB202190 inhibitor and stained with p-p38 antibody, and (D) UV stress +/- SP600125 inhibitor and stained with pJNK antibody. (E) BRAF WT or BRAF V600E mouse colon tissue stained with the BRAF V600E antibody.

**A B**

|  | EGF | + PD98059 |
| --- | --- | --- |
| HT29 | **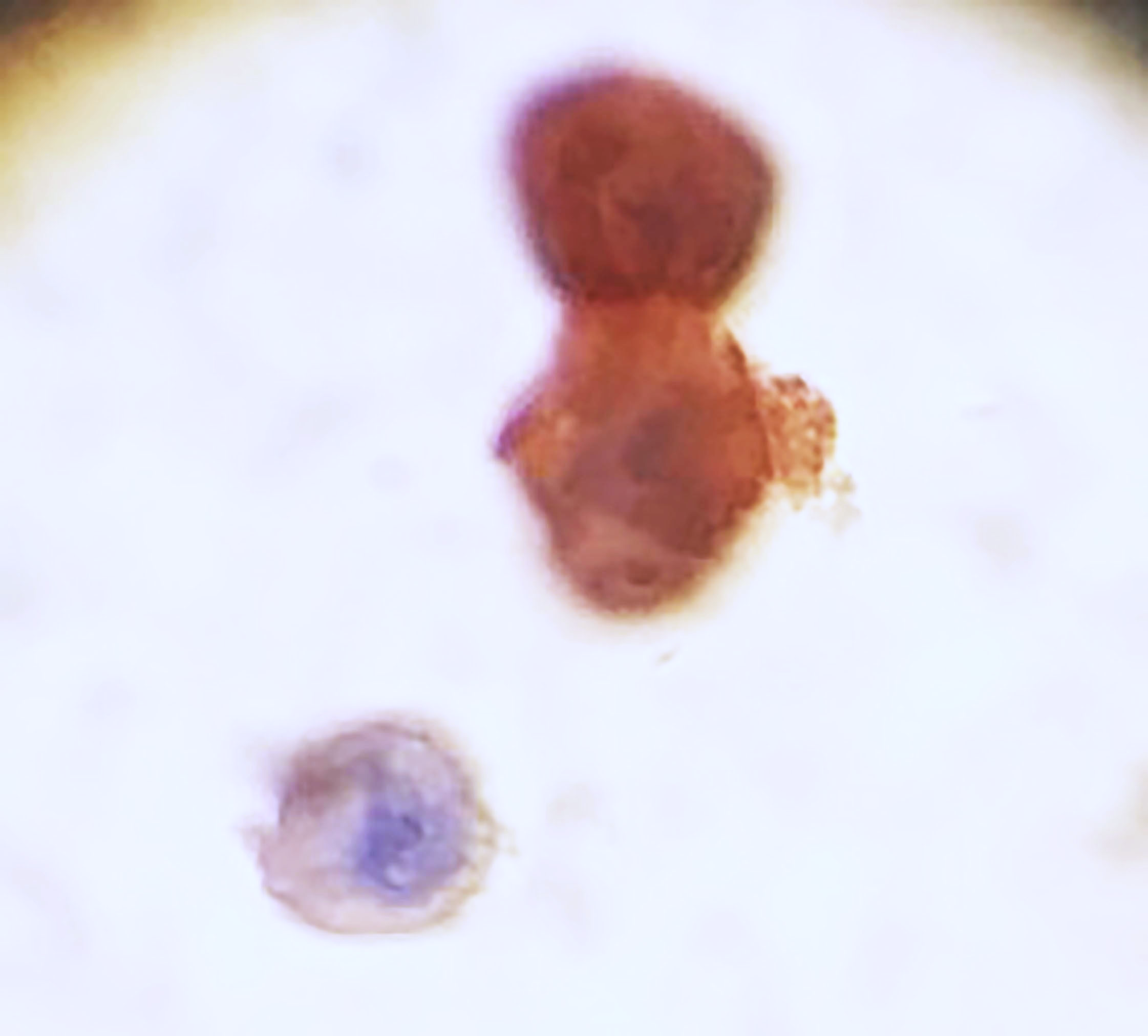**  10um 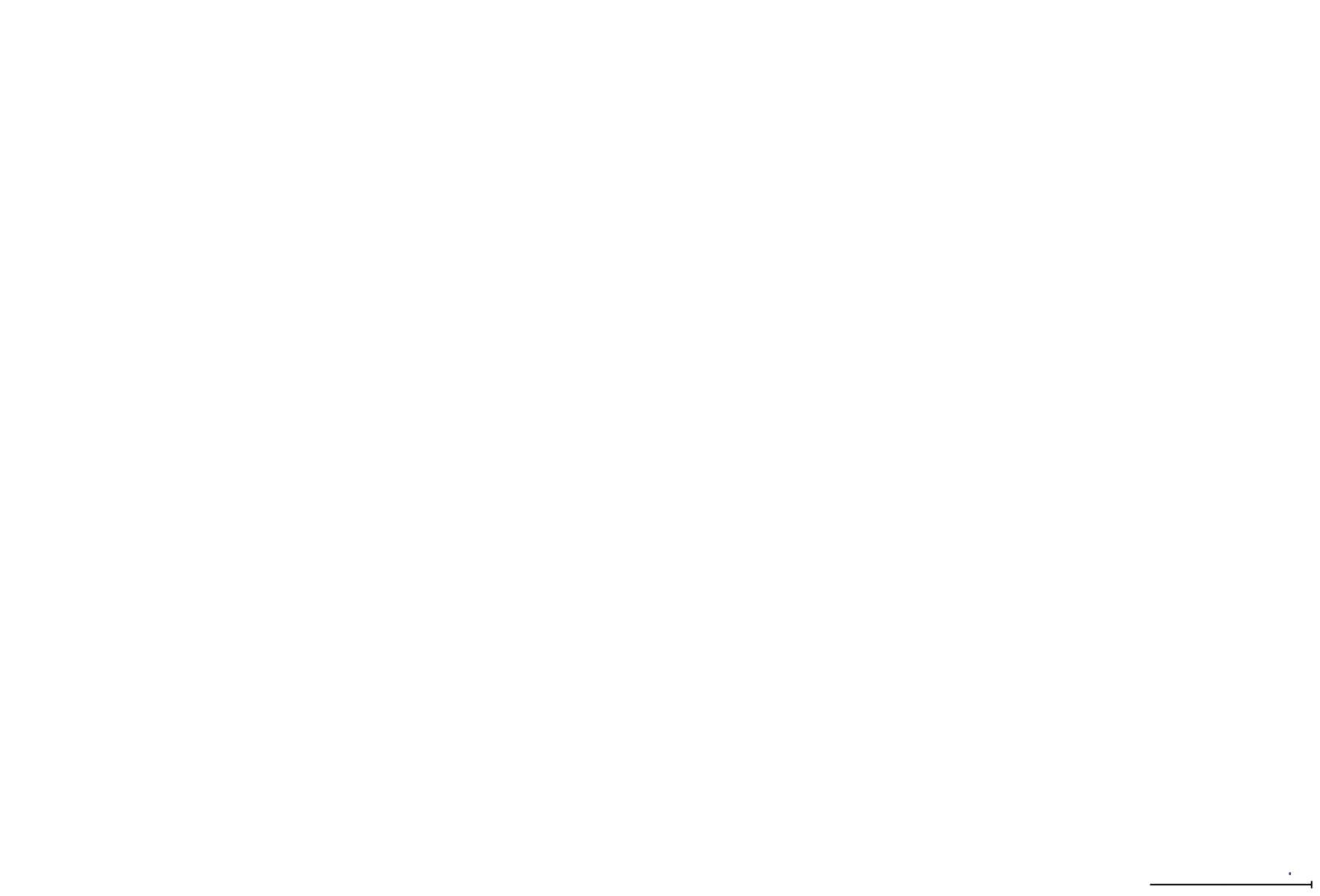 | **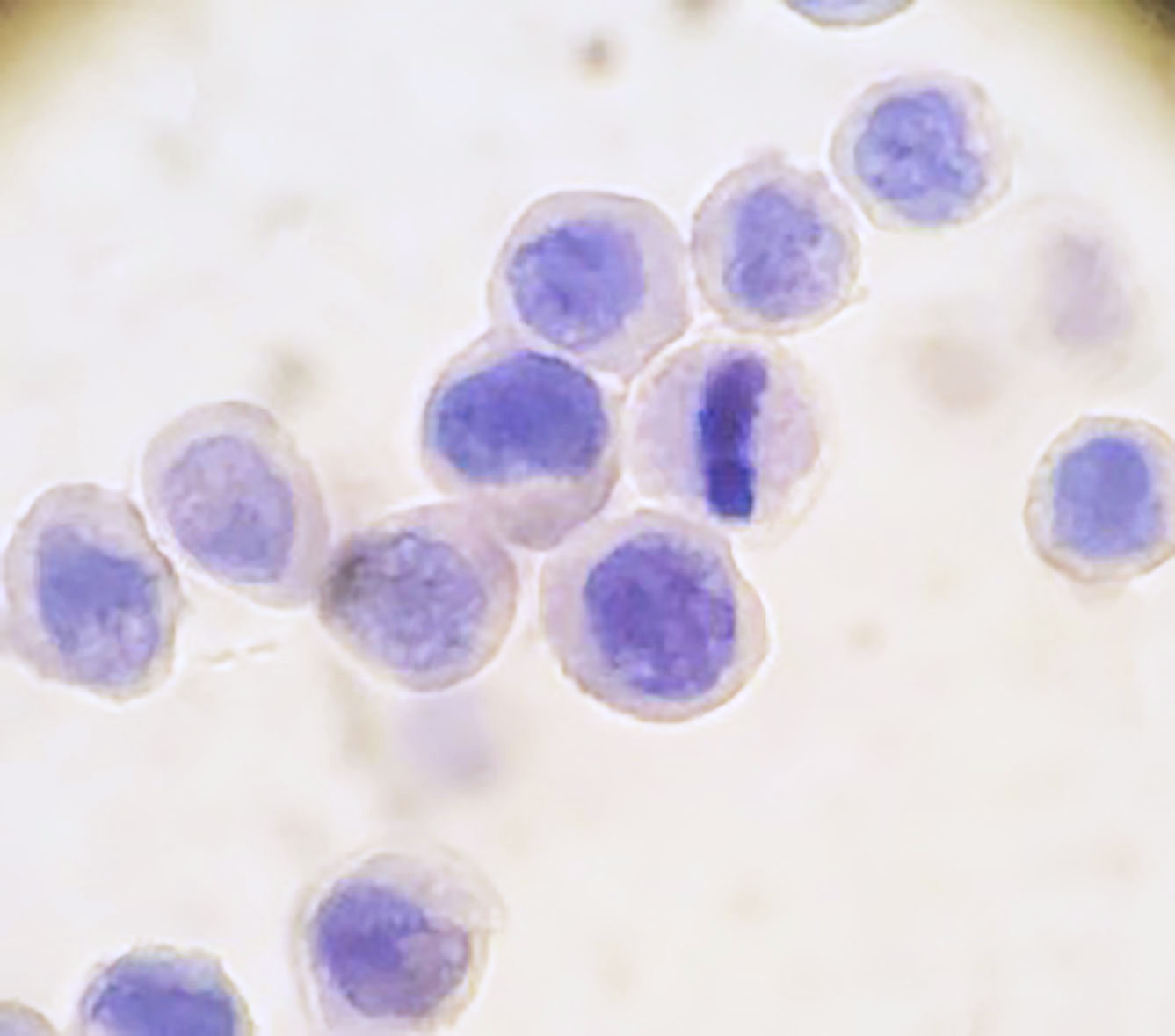**  10um 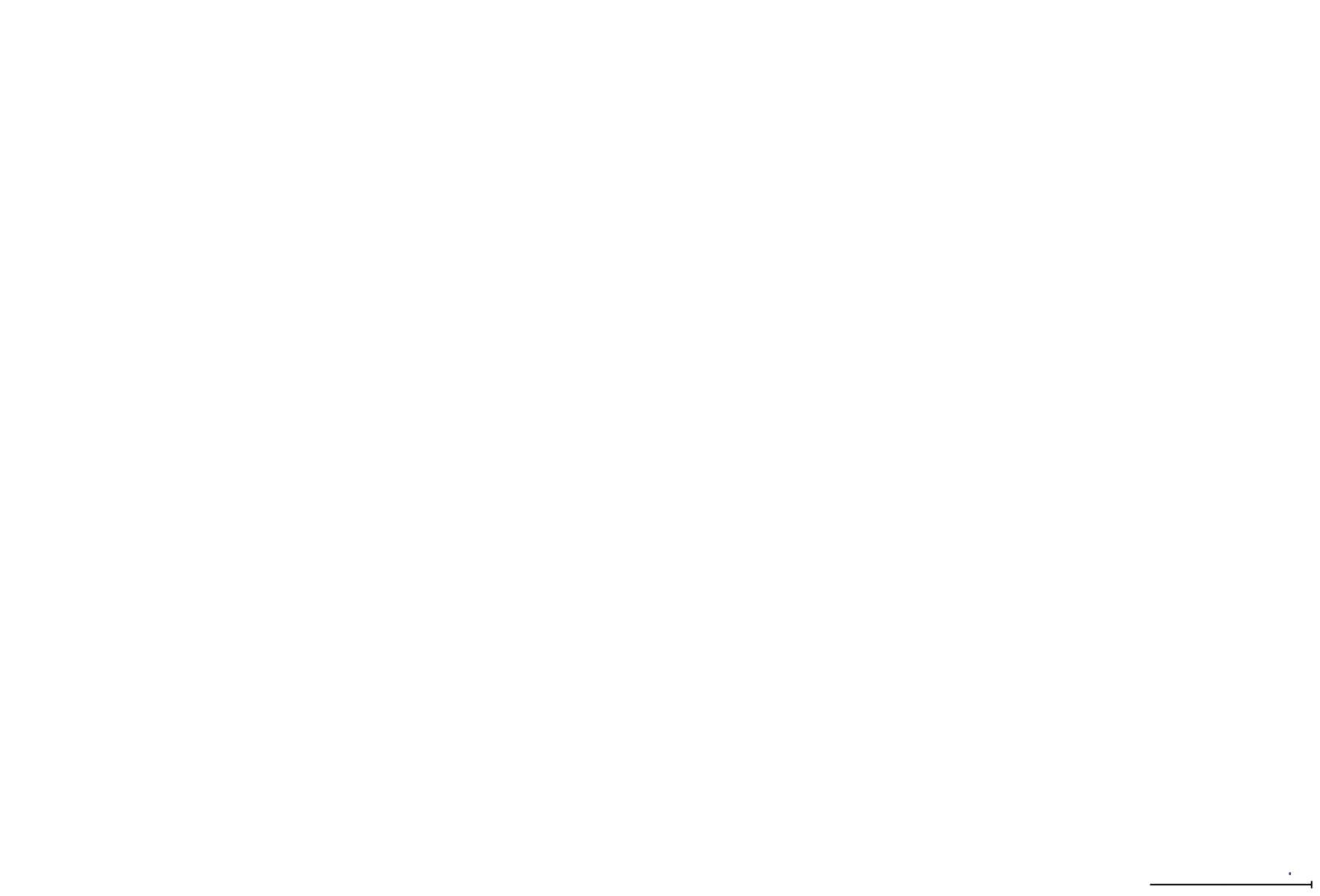 |
| T84 | **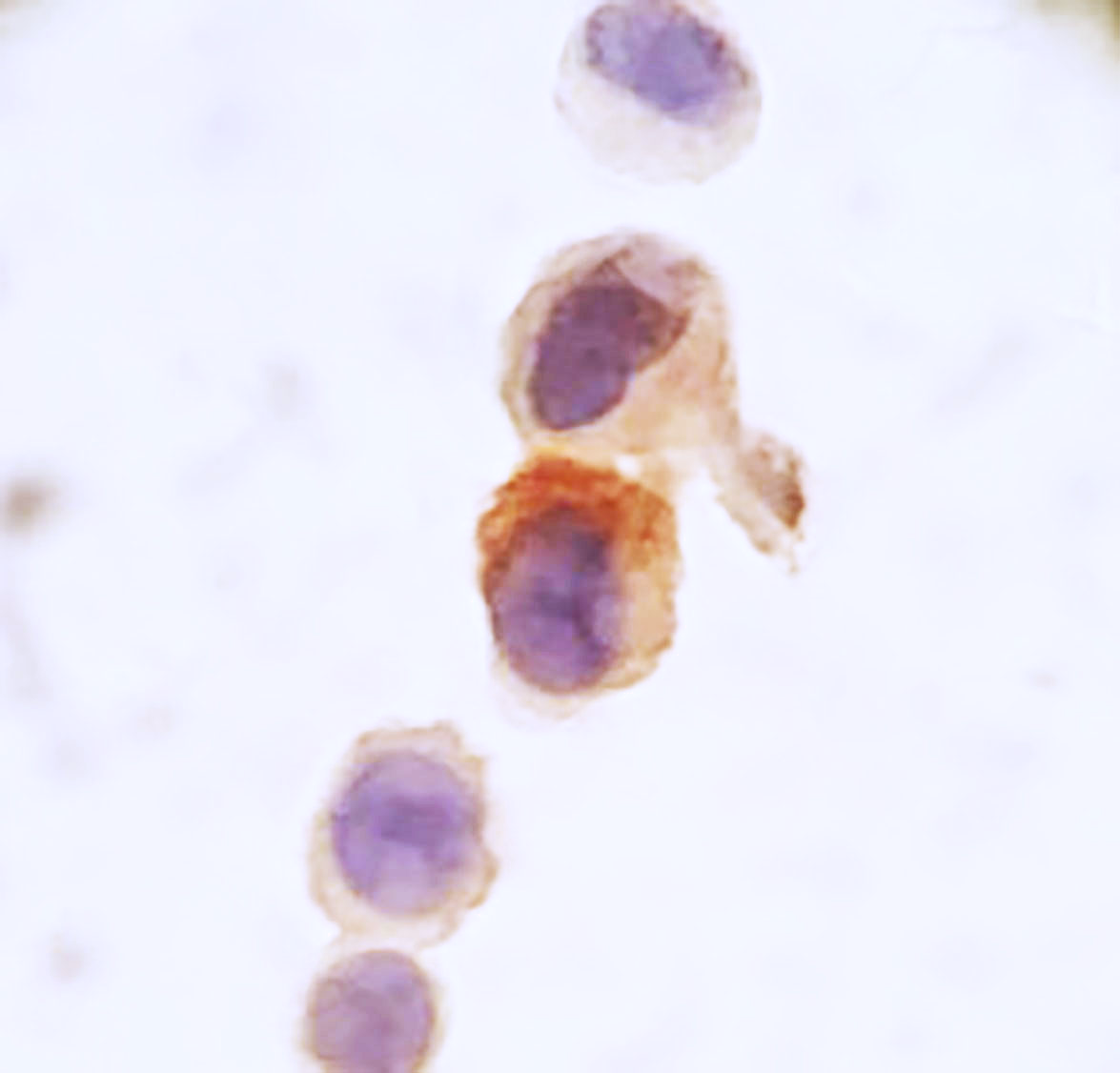** | **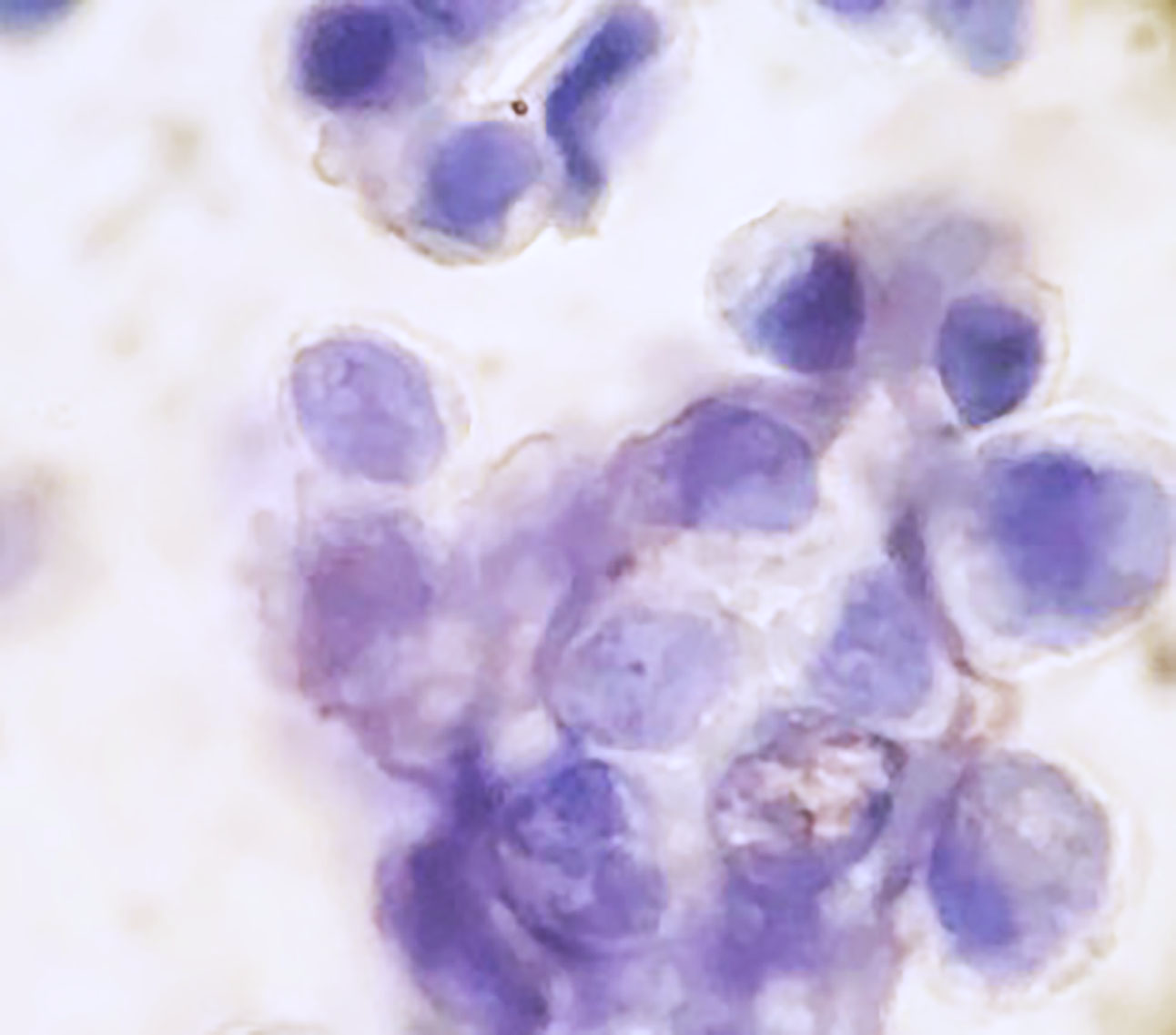** |

10um


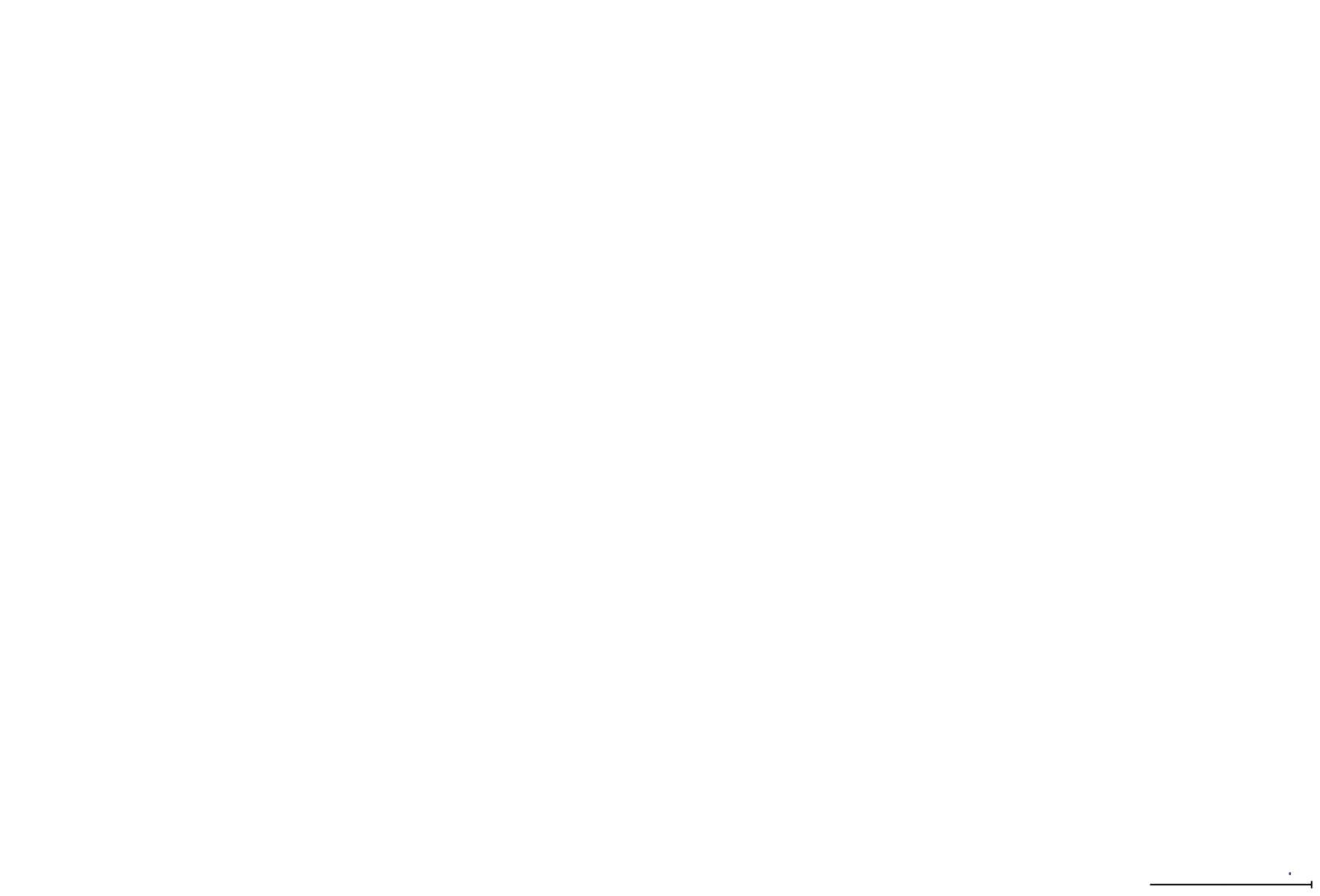


10um


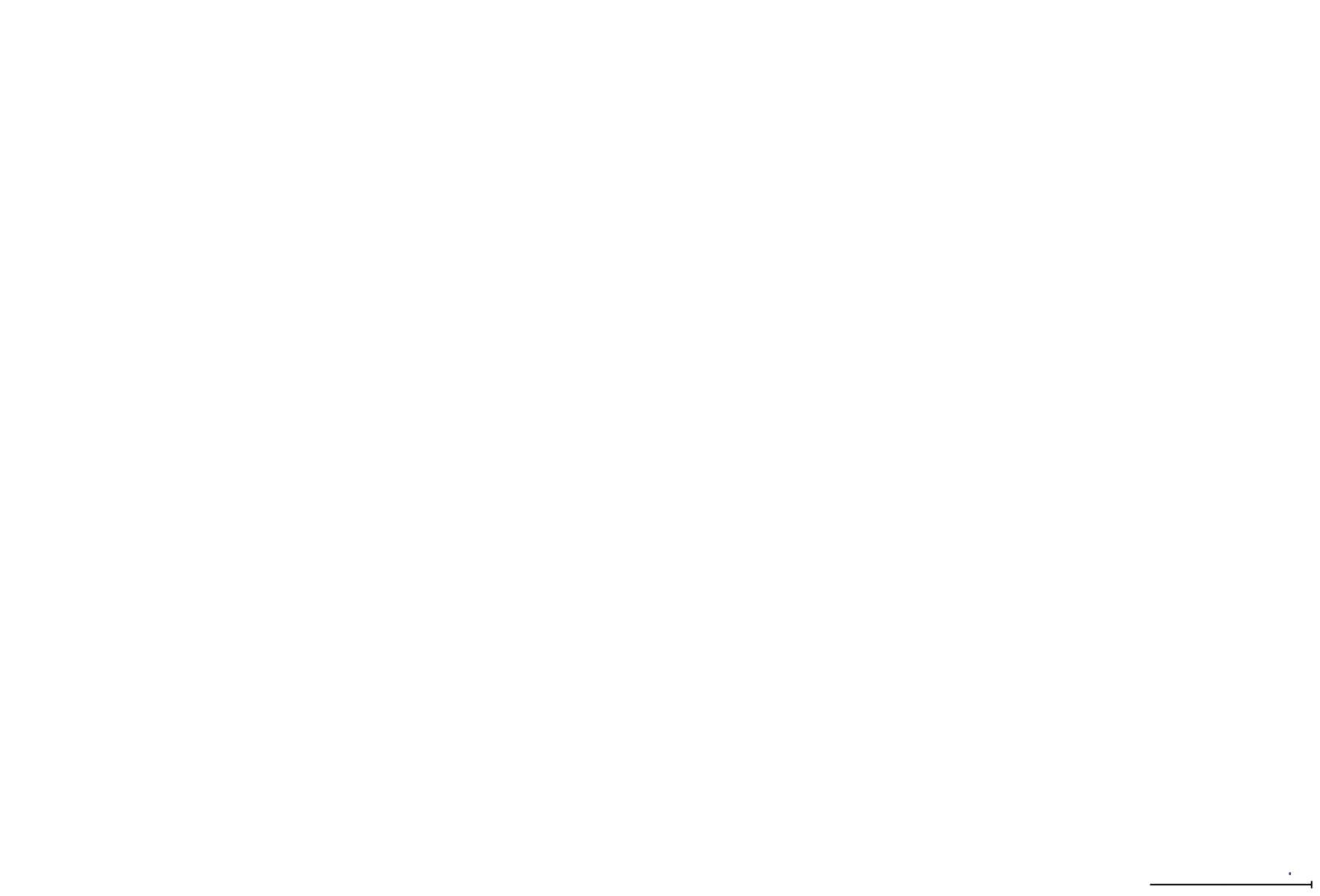


**C D**

10um


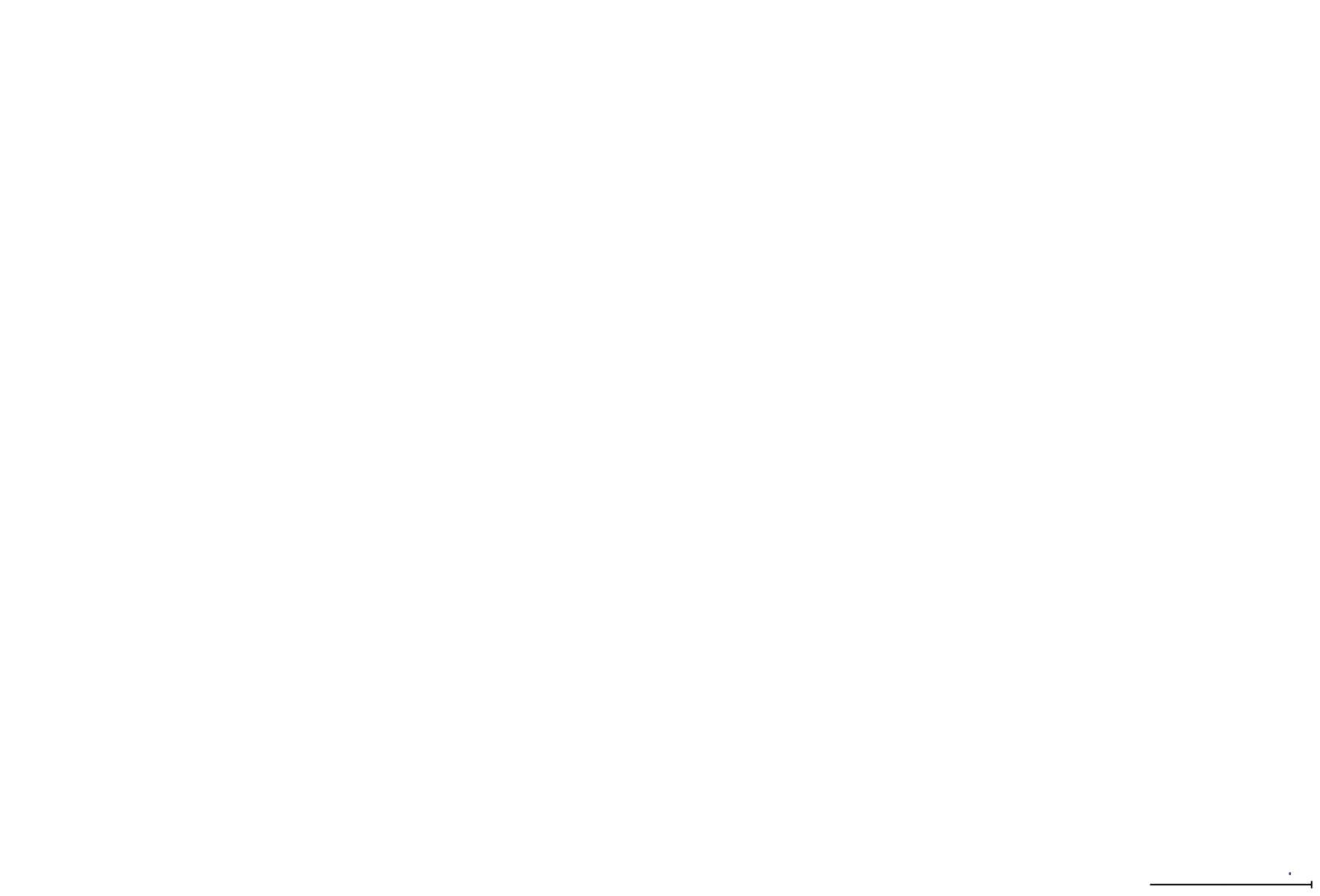


10um


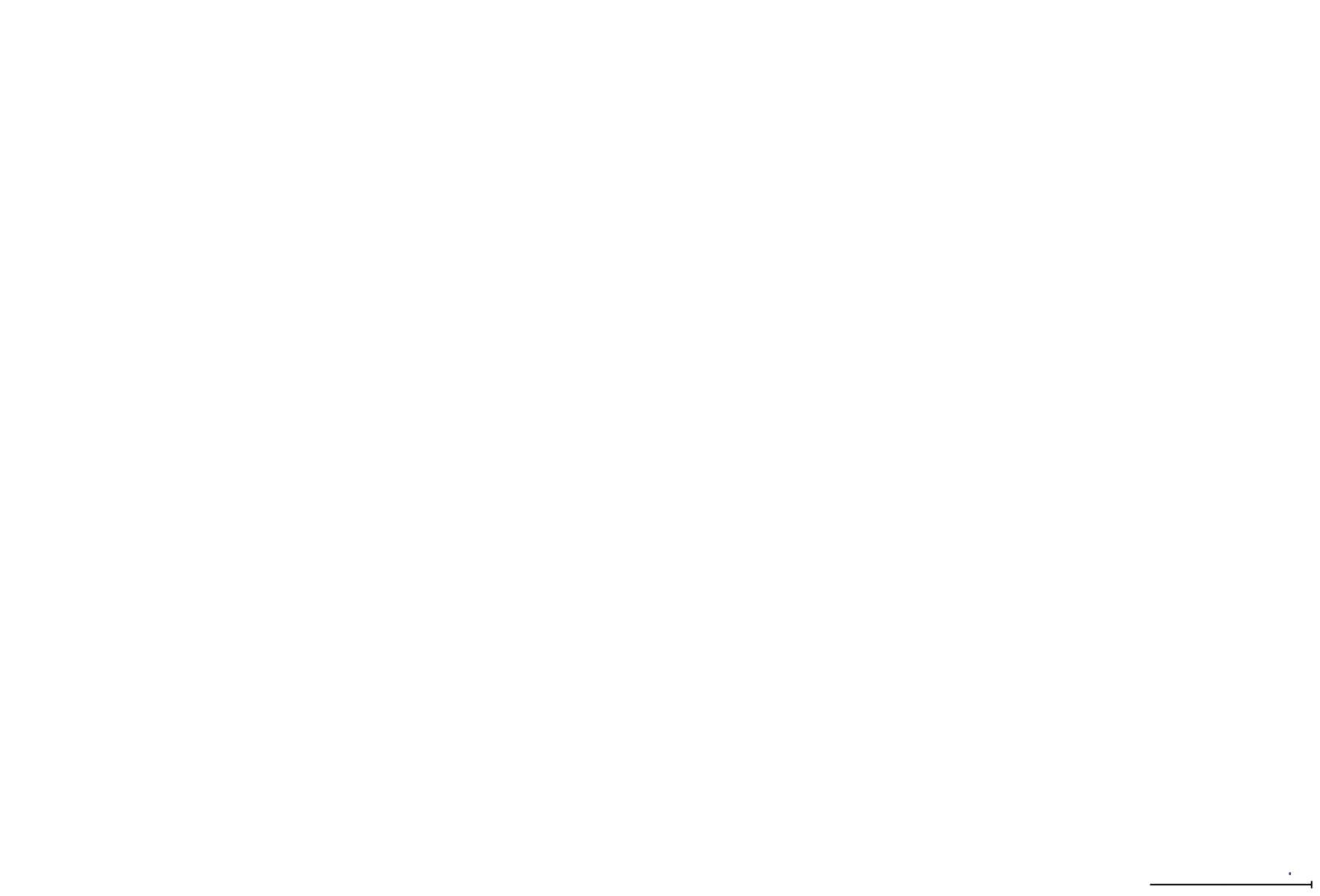


10um


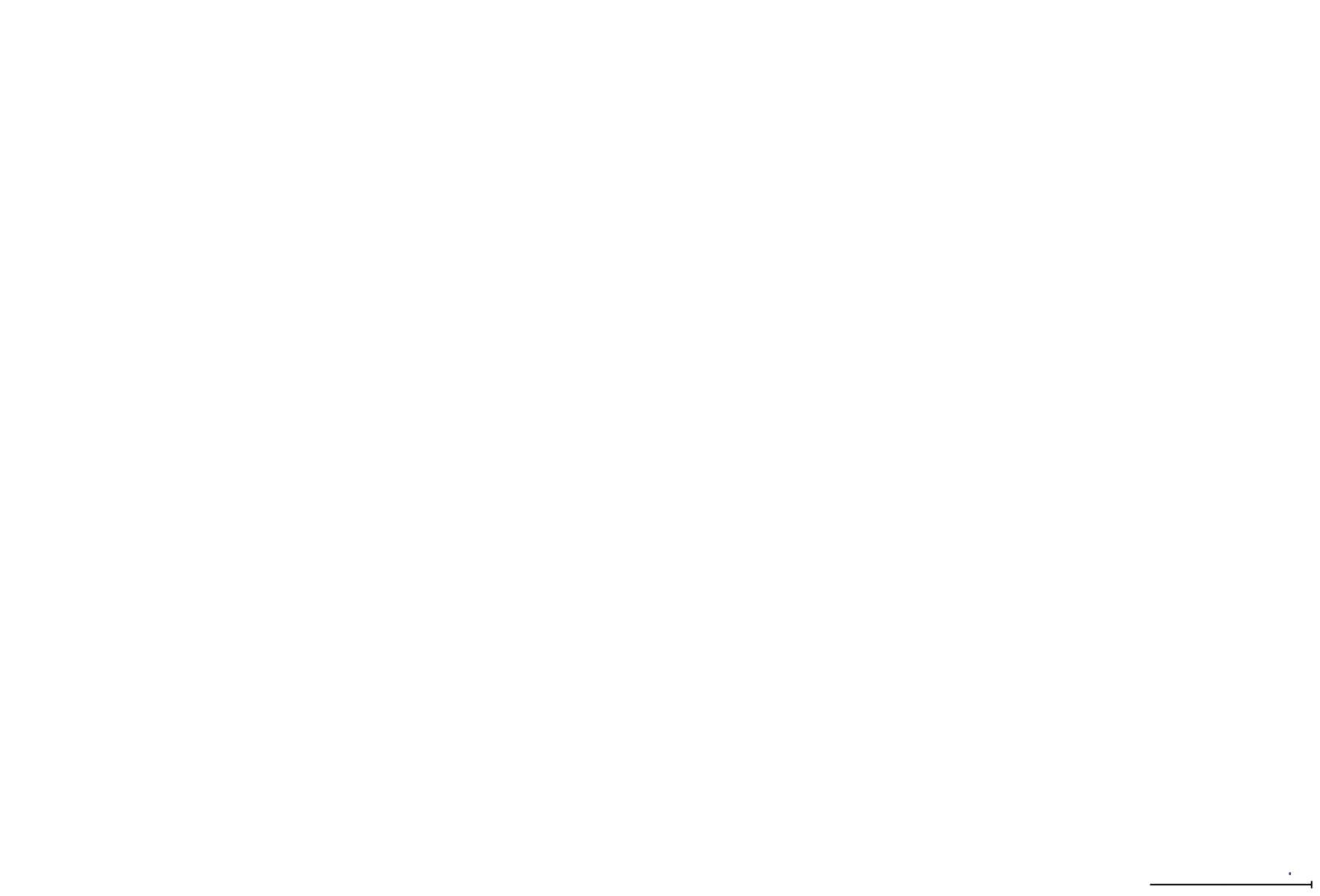


10um


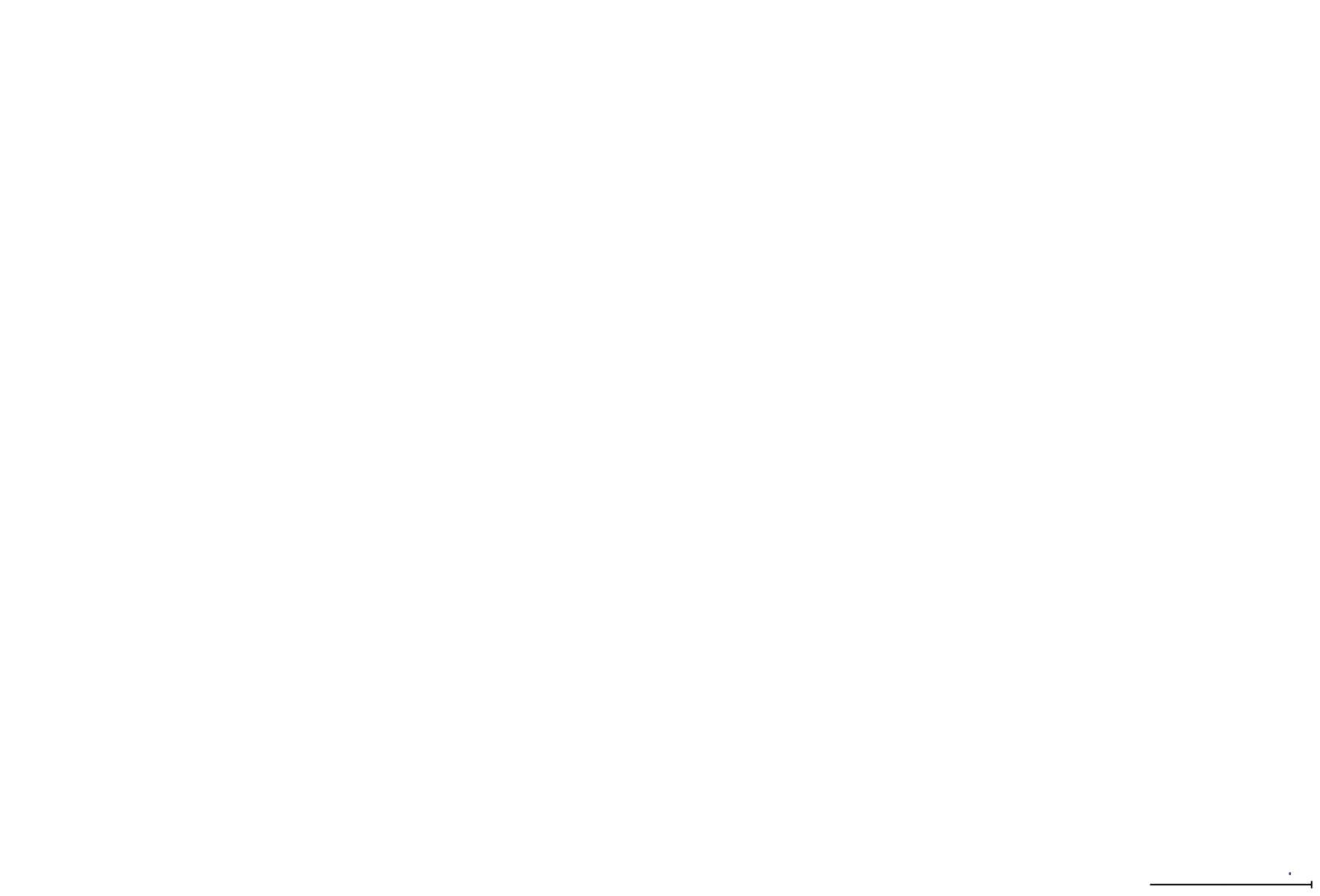


**E**

|  | UV | + SB202190 |
| --- | --- | --- |
| HT29 | **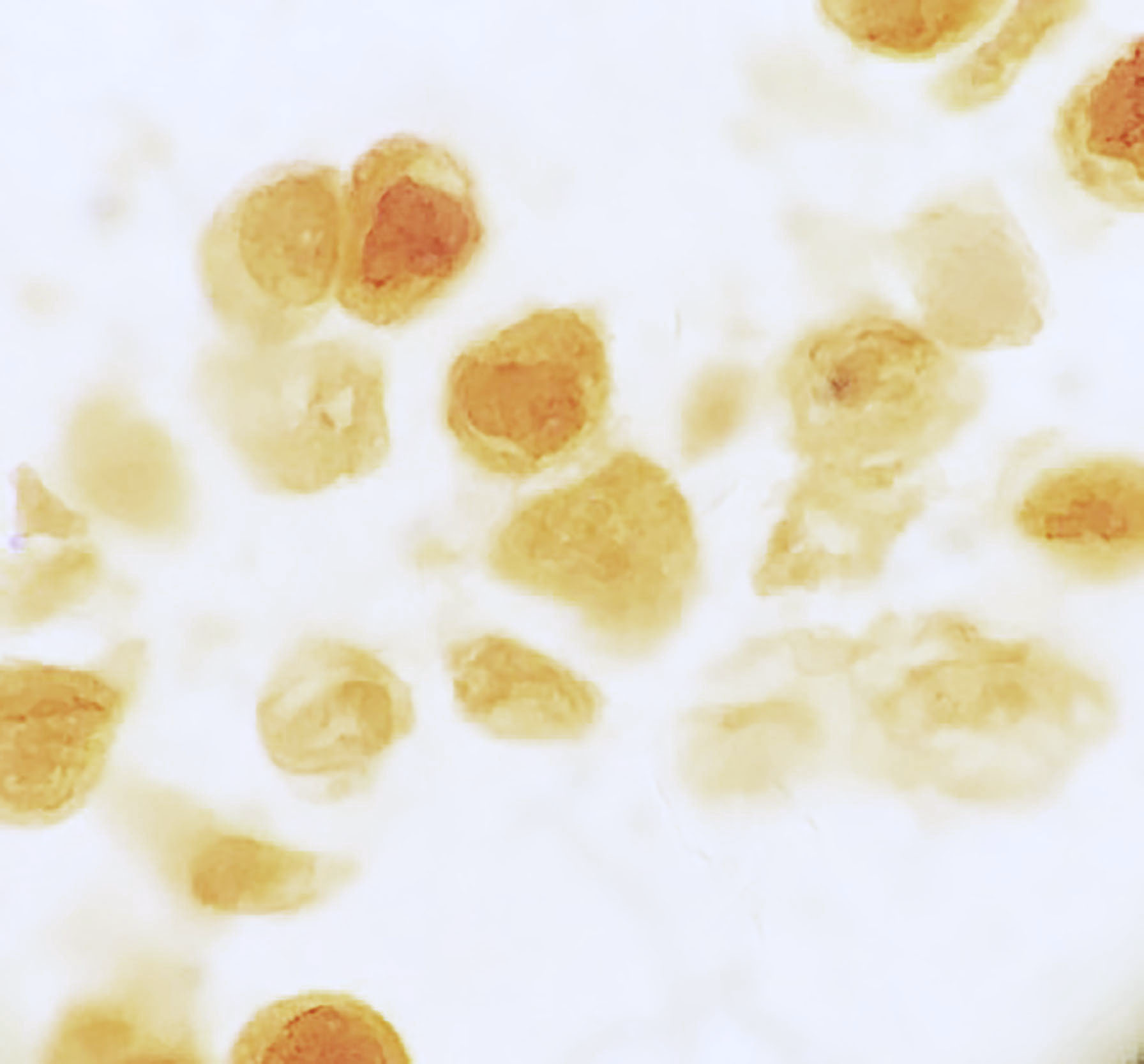**  10um 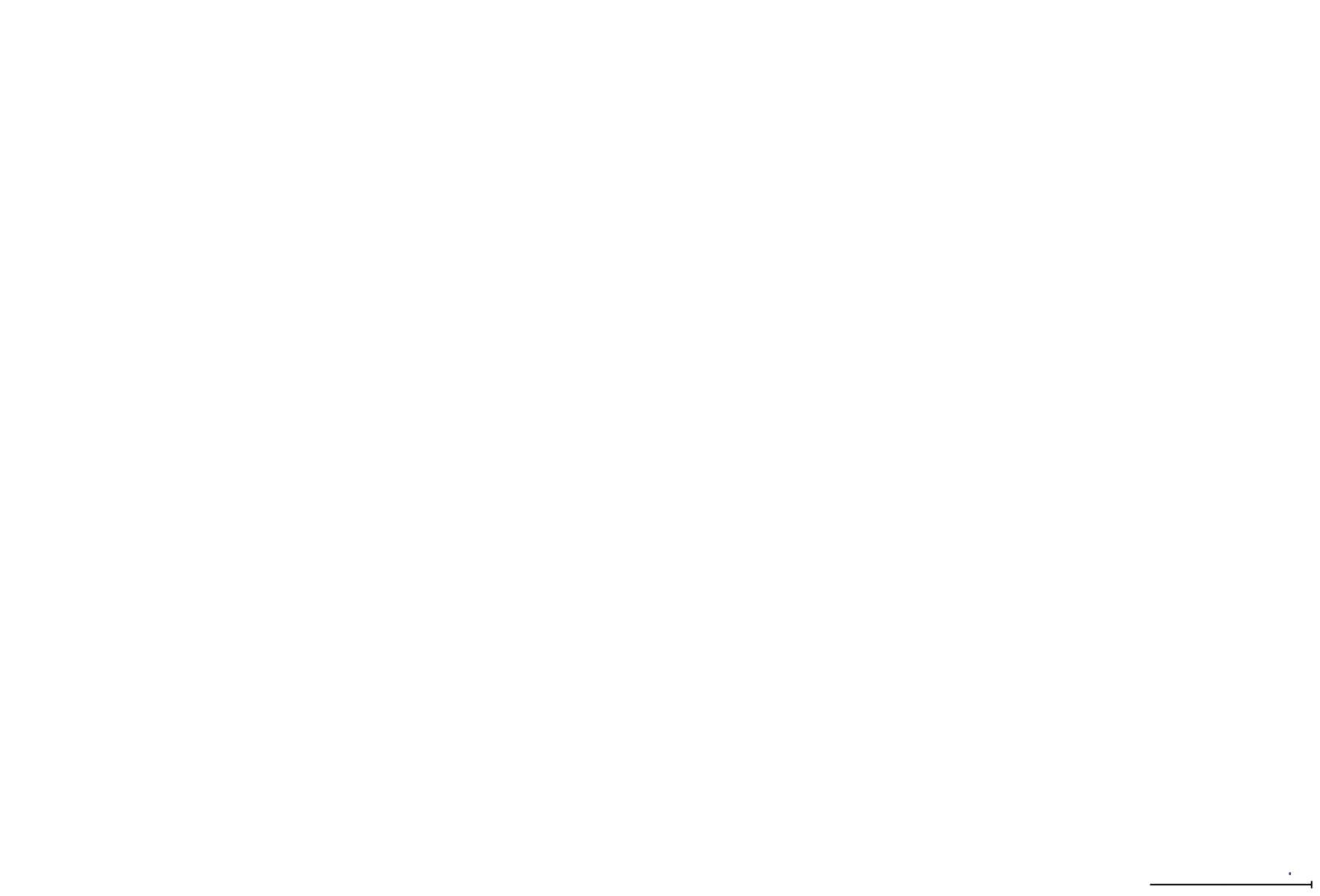 | **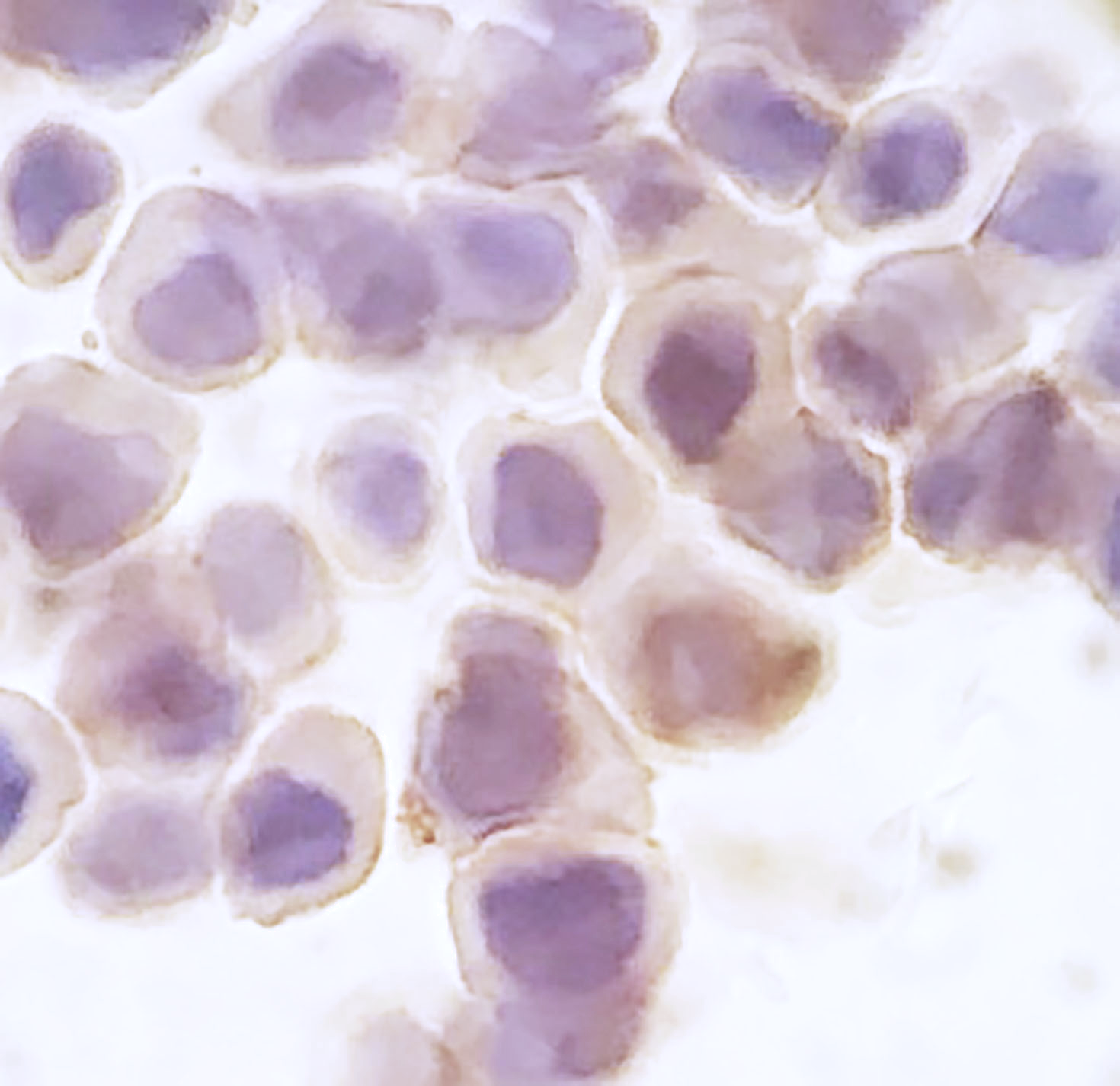**  10um 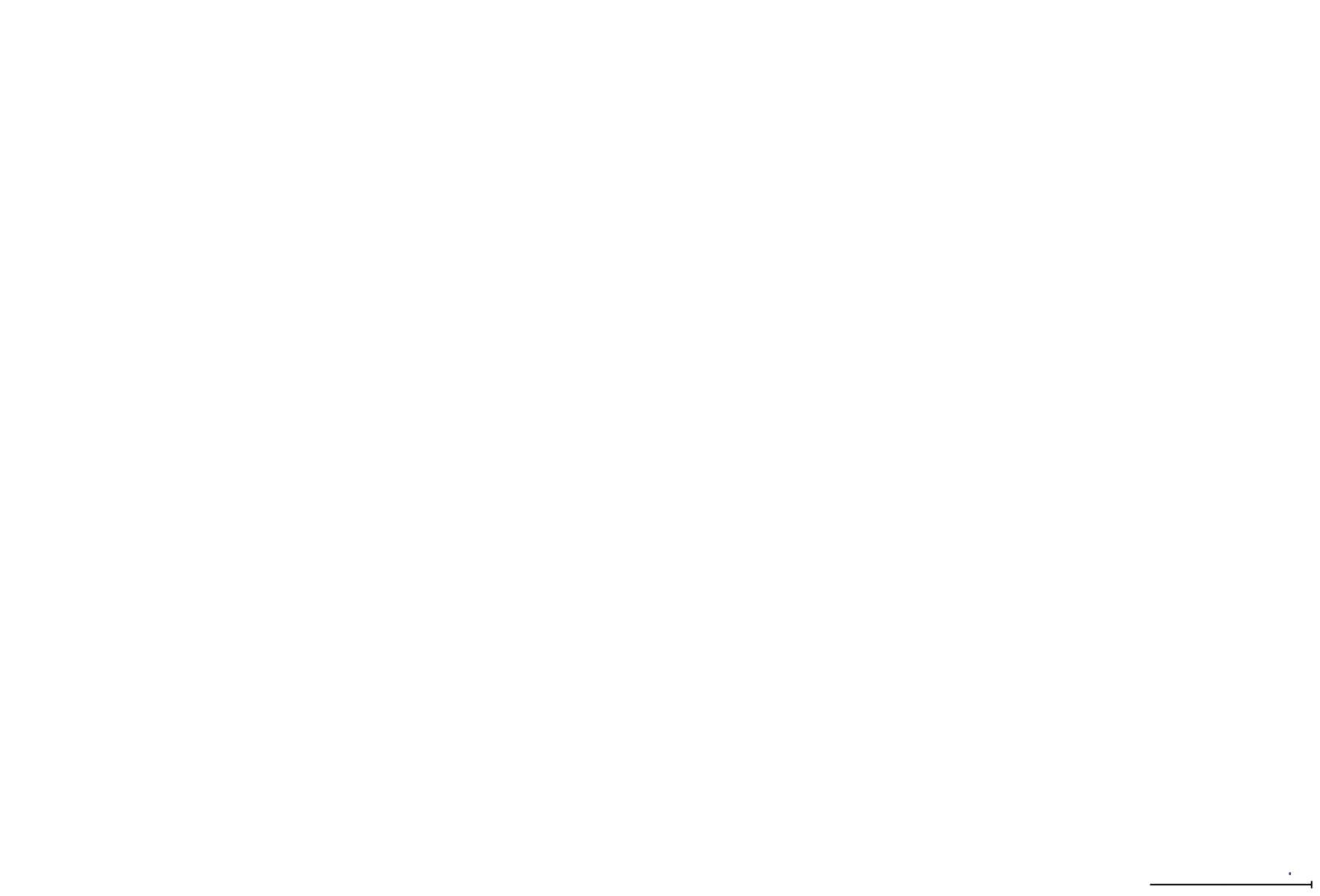 |
| T84 | **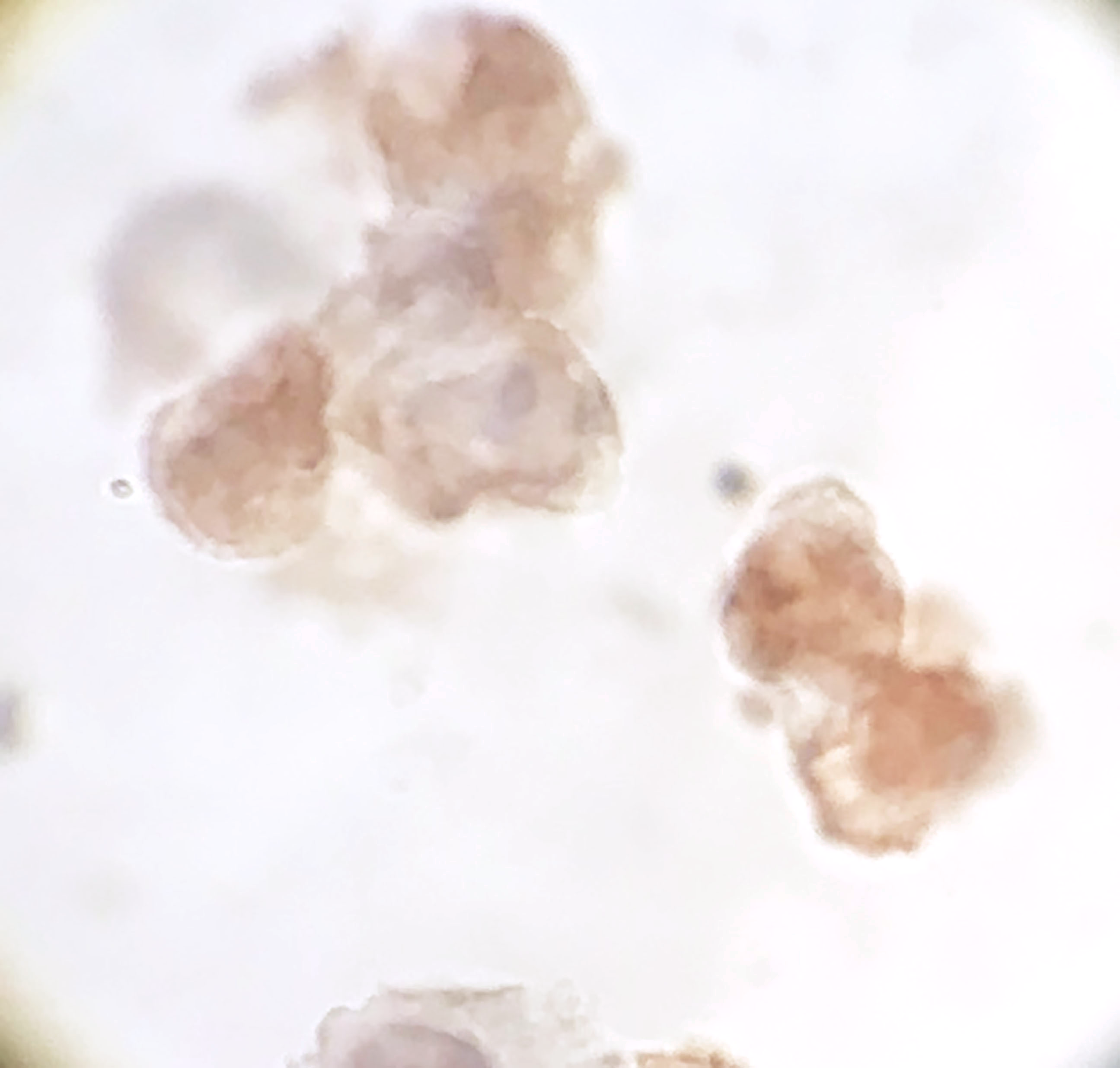** | **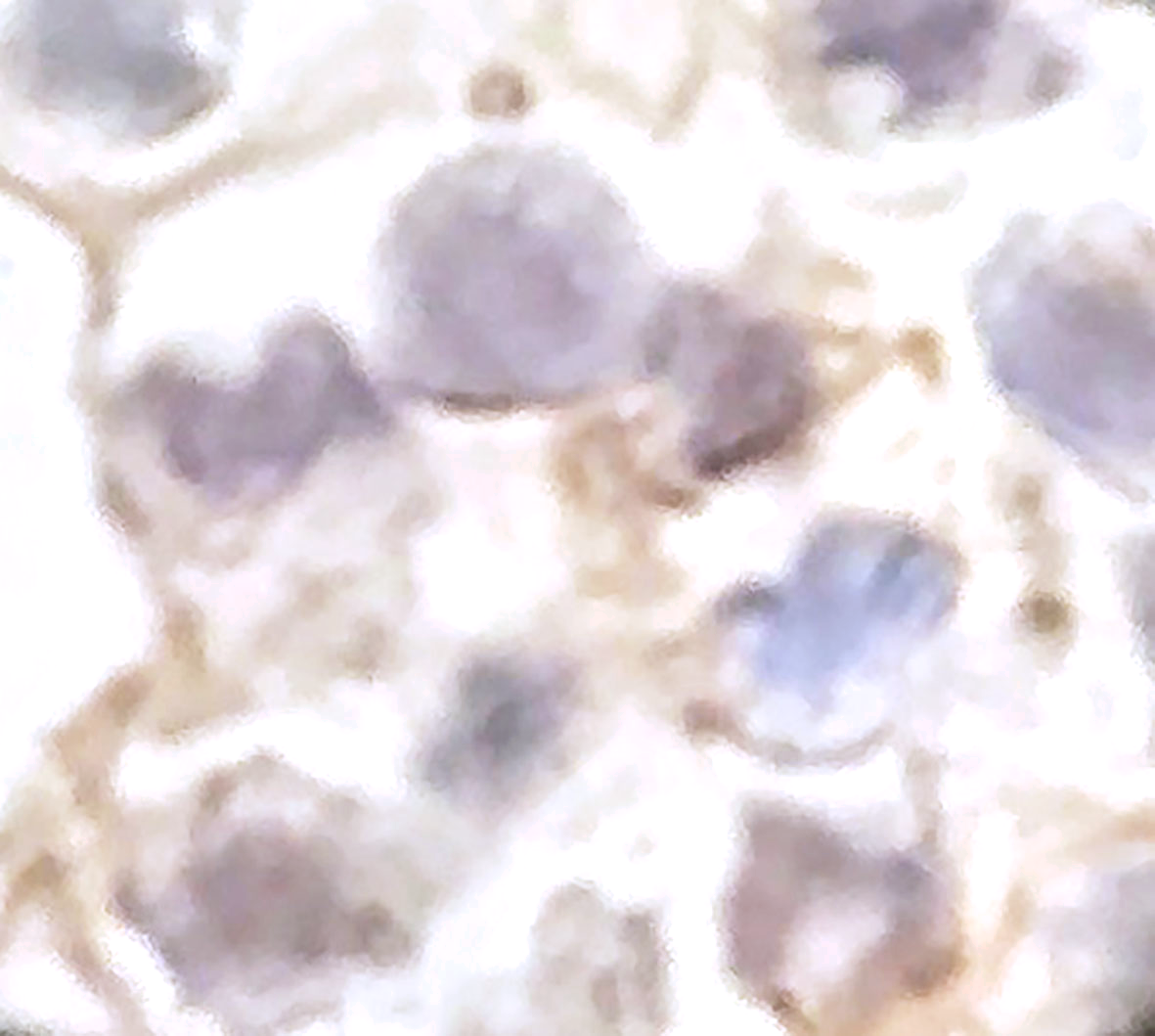** |

|  | UV | + SP600125 |
| --- | --- | --- |
| HT29 | **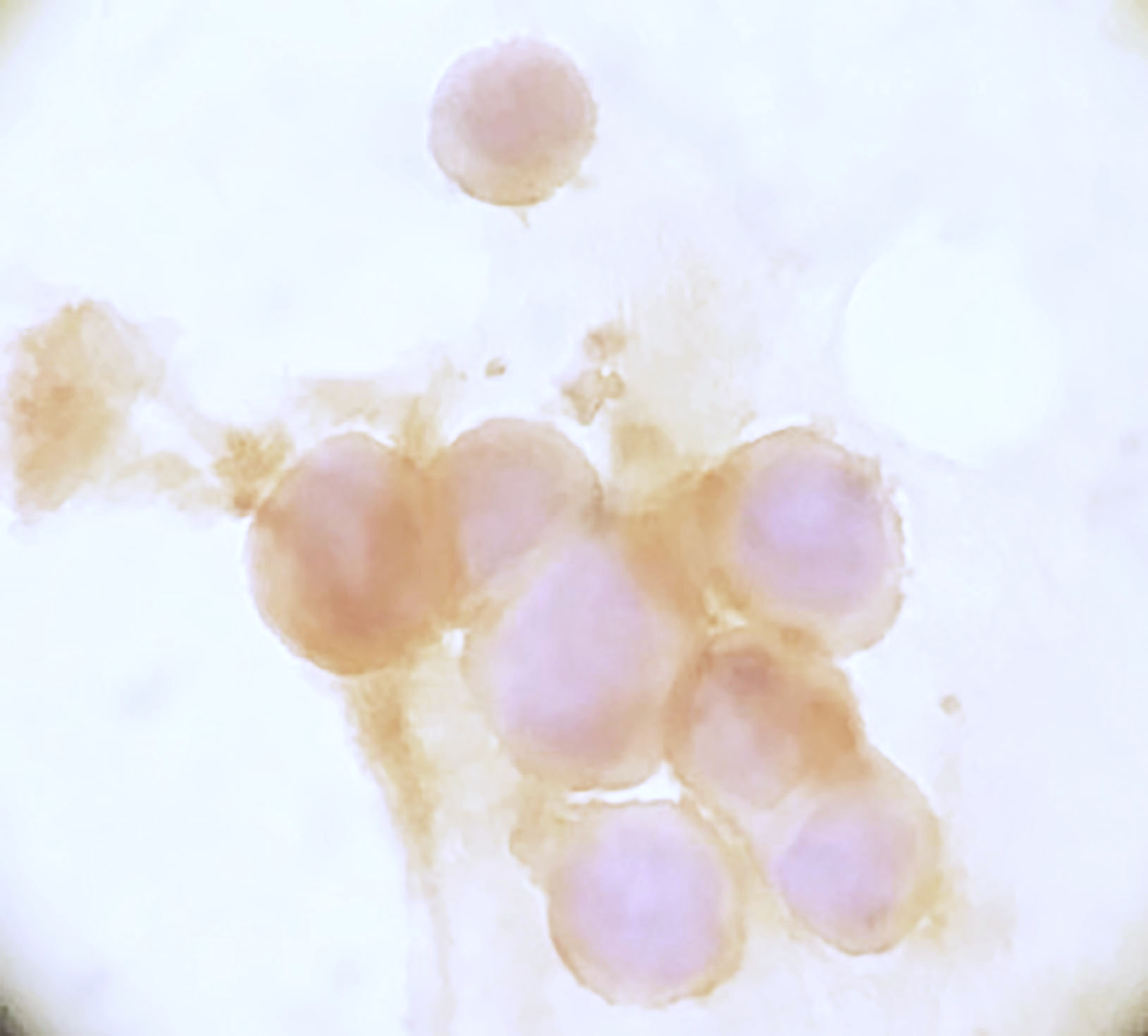**  10um 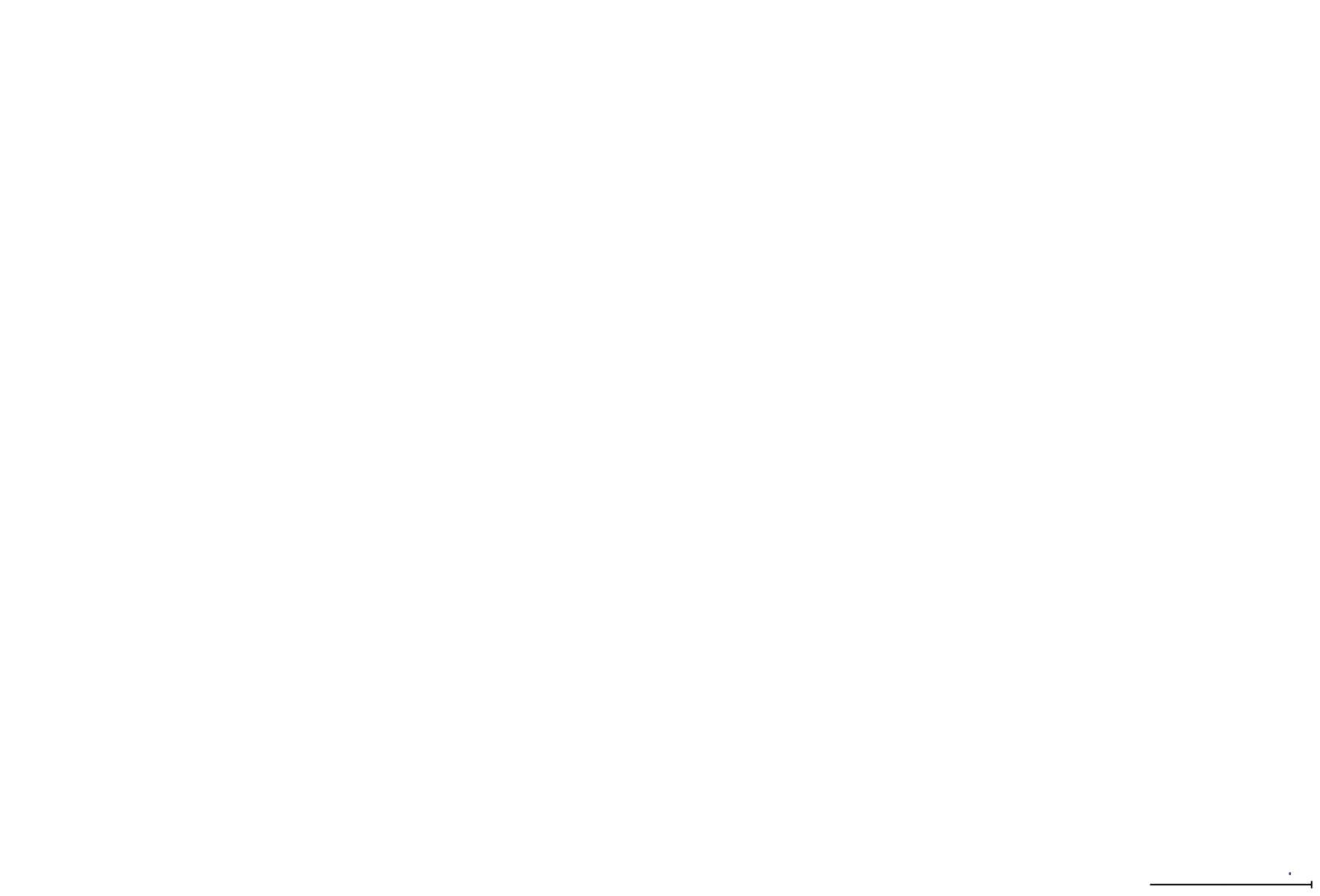 | **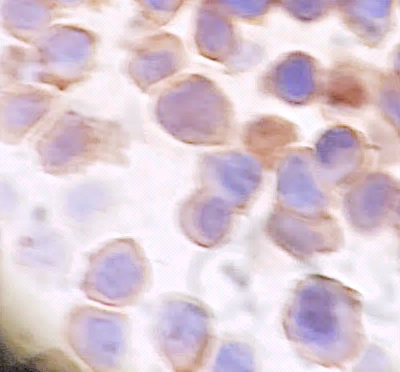**  10um 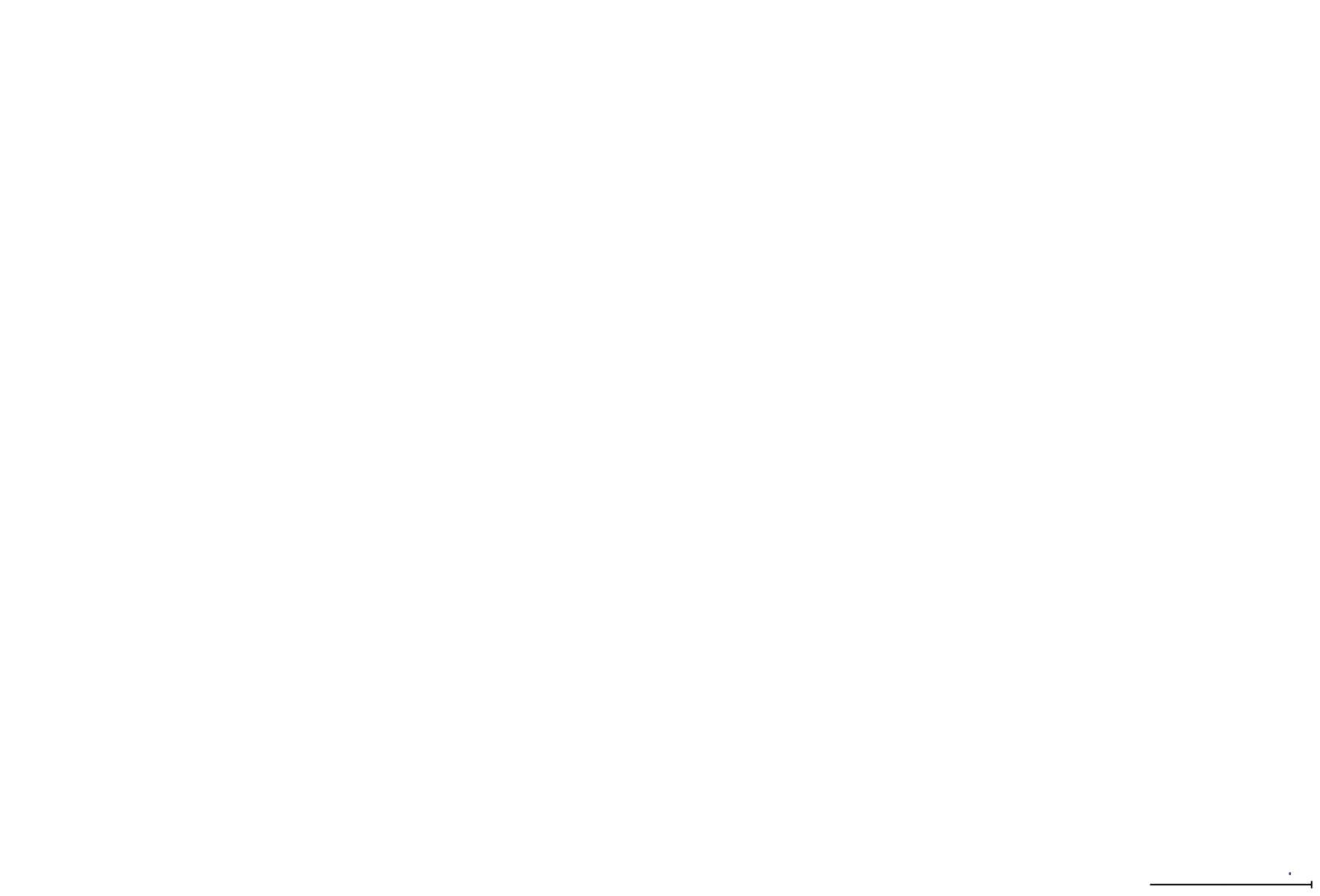 |
| T84 | **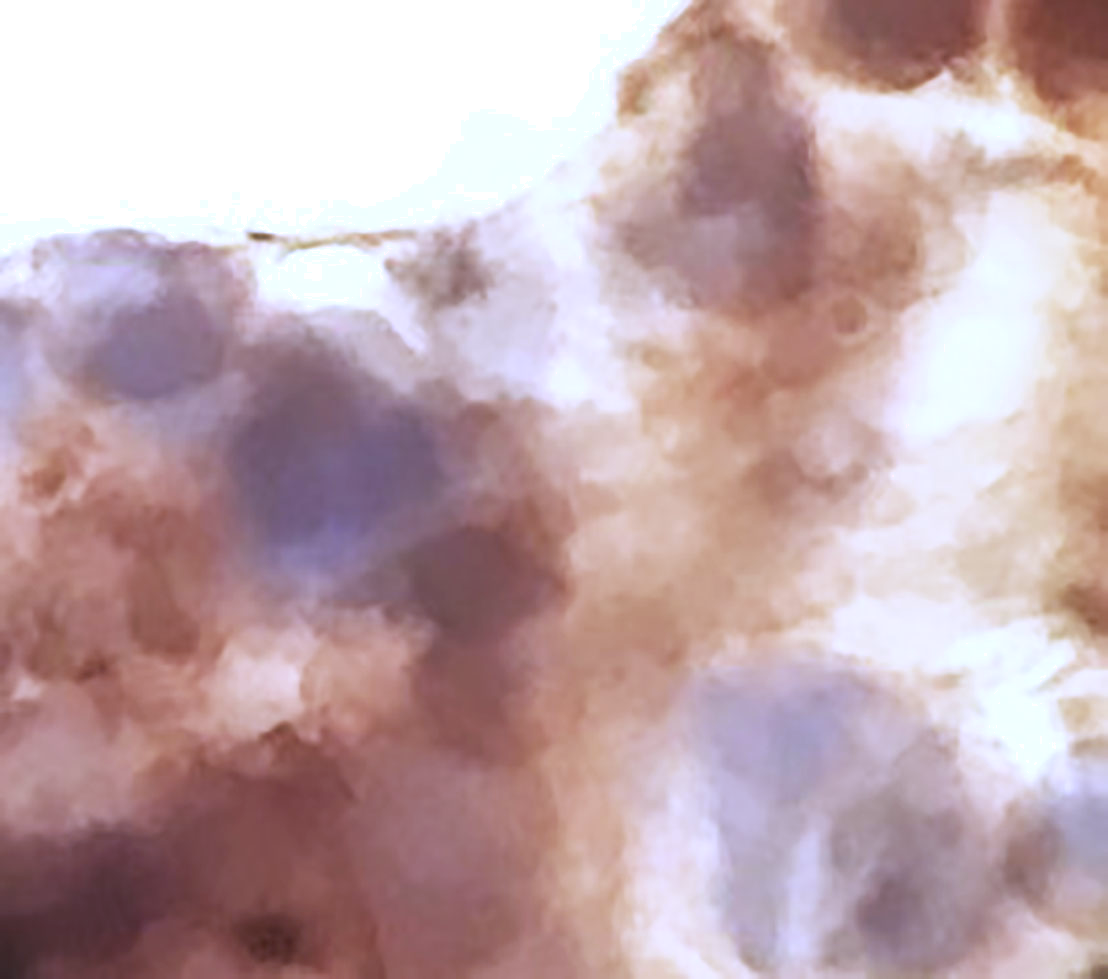** | **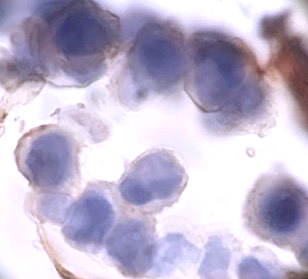** |

| BRAF WT | | BRAF V600E 1 copy | BRAF V600E 2 copies | |
| --- | --- | --- | --- | --- |
| **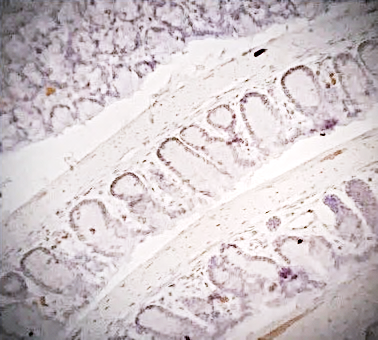**  100um 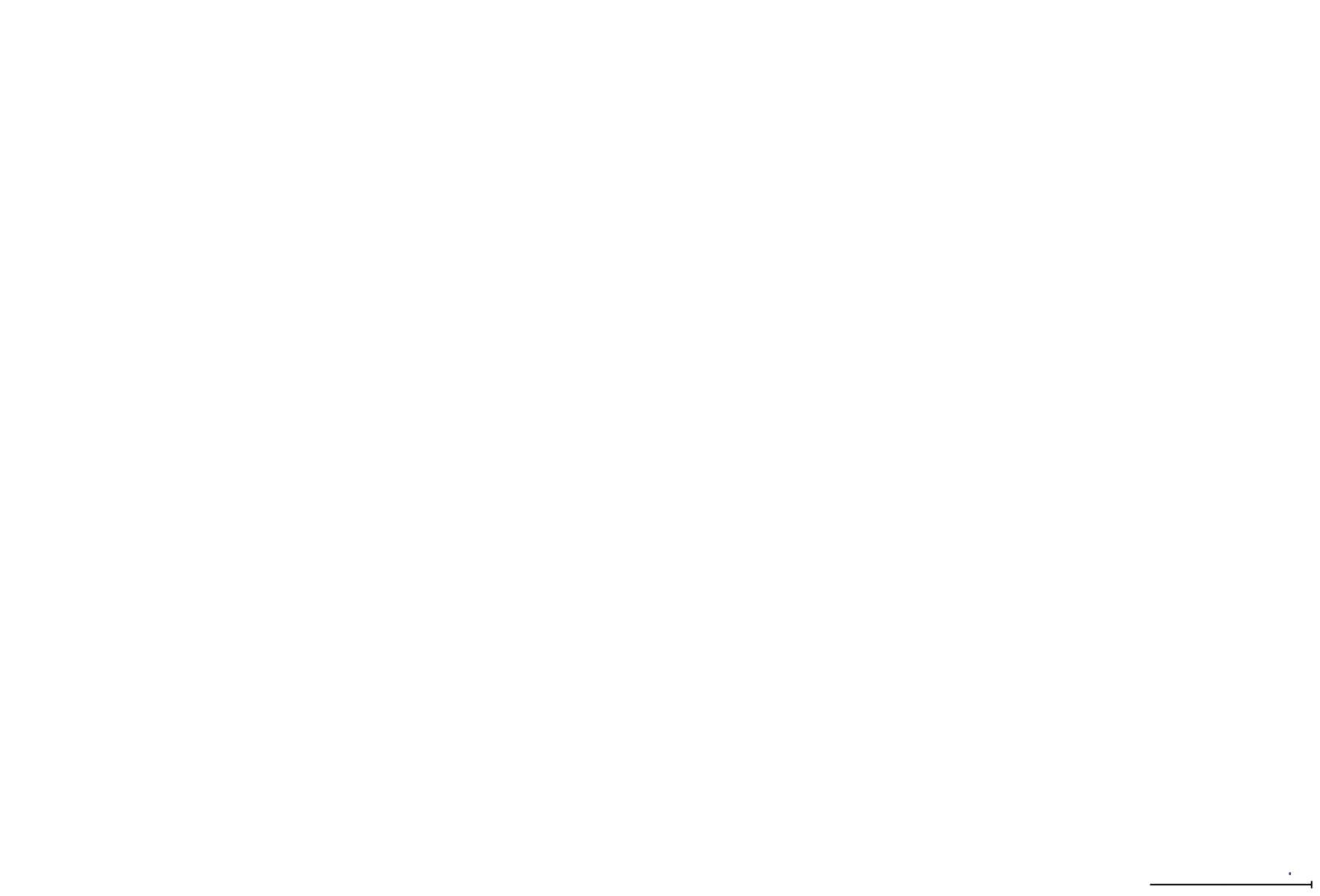 | **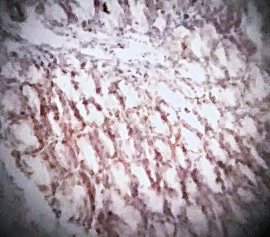**  100um 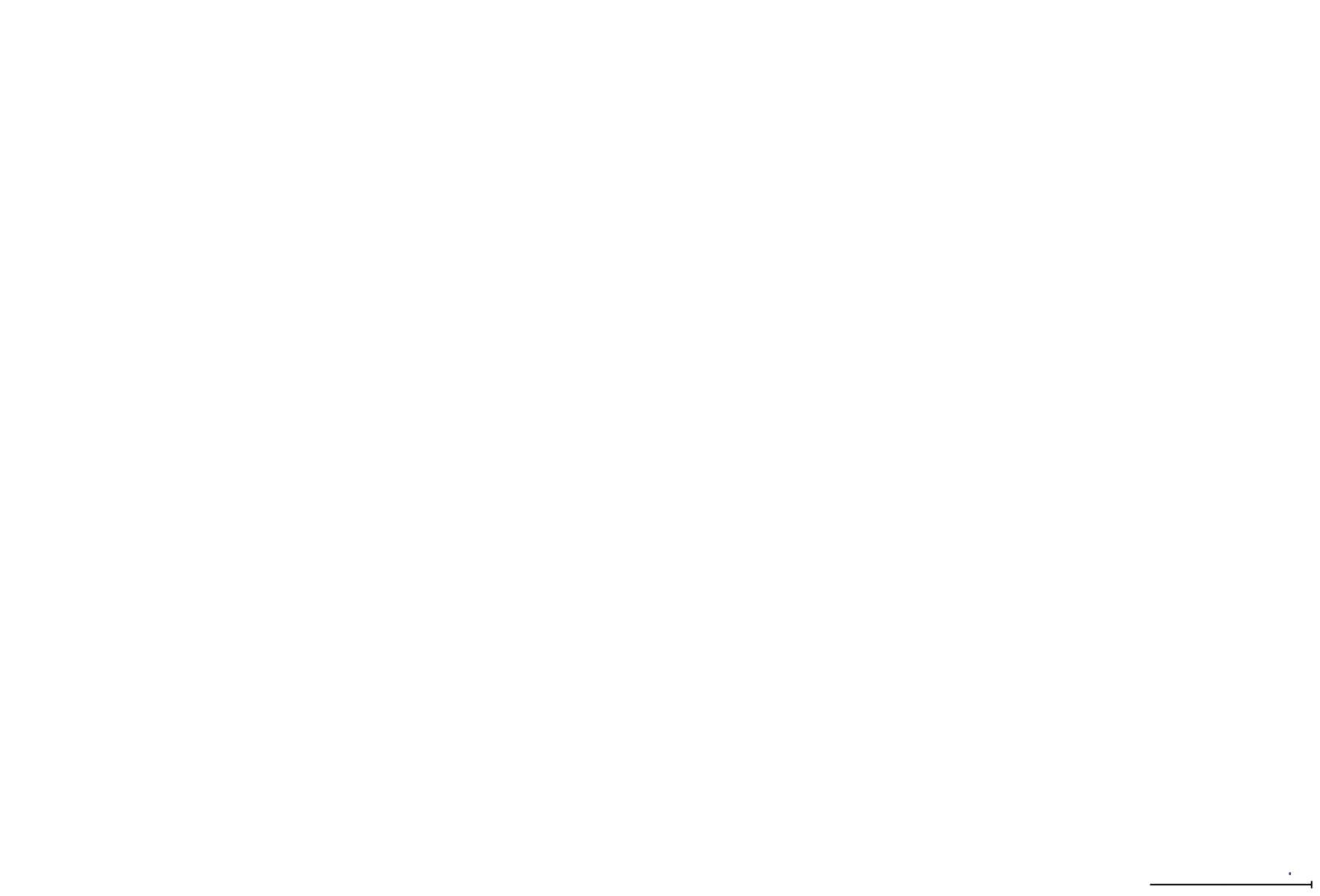 | | **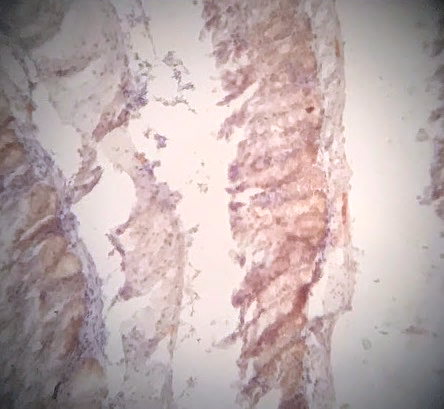**  100um 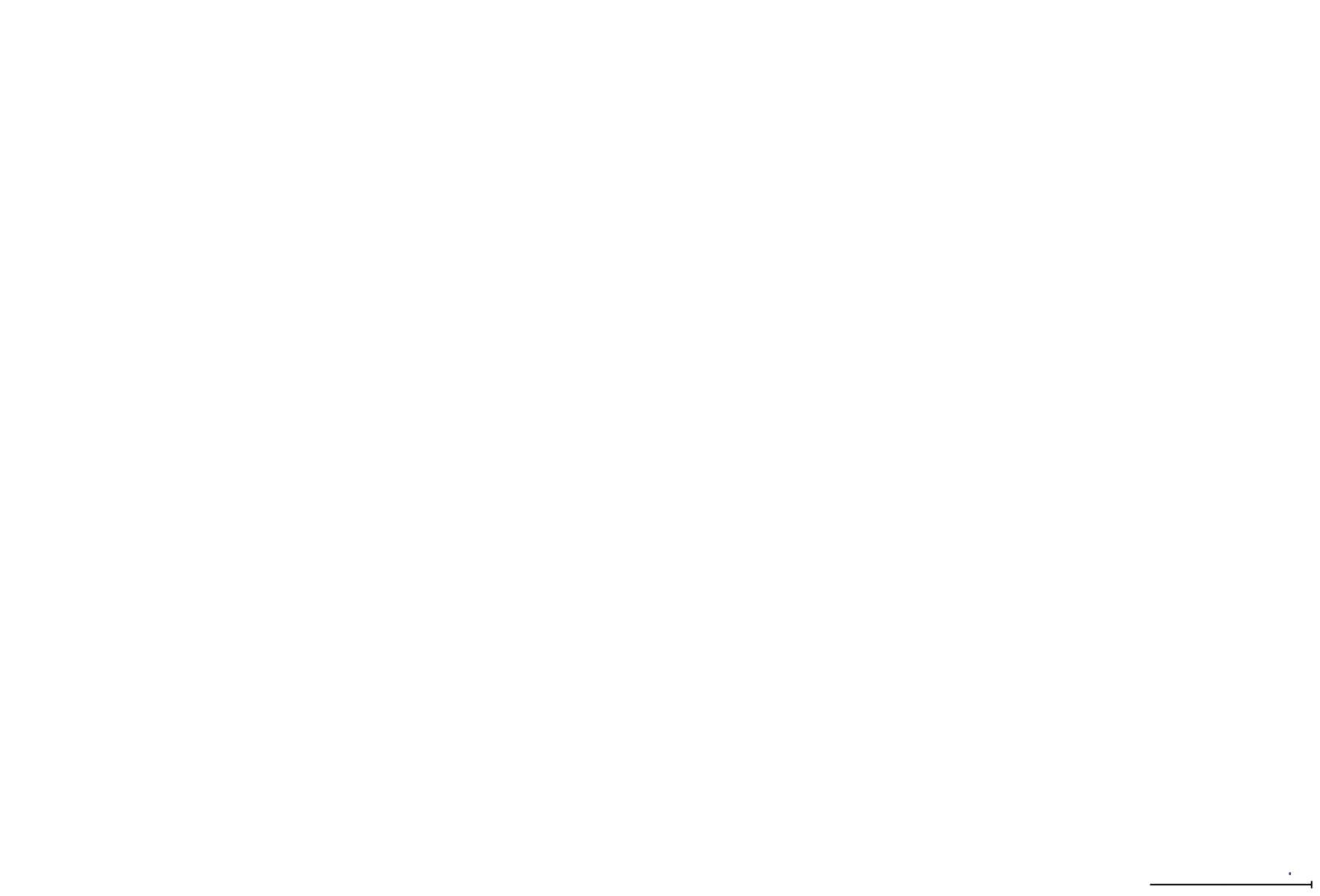 |  |

**Figure S3. Combined nuclear pERK/p-p38 score stratifies CSS in MMR competent BRAF mutant stage III CRC patients (n=165).**Kaplan Meier curves showing association between the combined nuclear pERK/p-p38 score and CSS in MMR competent BRAF mutant patients with (A) Stage I, (B) Stage II or (C) Stage 3 CRC.


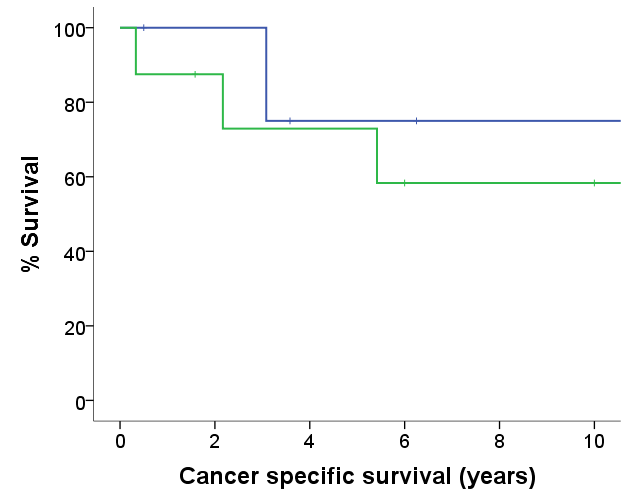
 **A**

**TNM-Stage I**

Both weak or one strong

Both strong

HR 1.84 95% CI 0.19-17.94 log rank p=0.593

| Number of  patients at risk |  |  |  |  |  |  |
| --- | --- | --- | --- | --- | --- | --- |
| Low | 5 (0) | 4 (1) | 2 (2) | 2 (2) | 1 (3) | 1 (3) |
| High | 8 (0) | 6 (1) | 5 (1) | 4 (1) | 3 (2) | 3 (2) |

**B**

**TNM-Stage II**

Both weak or one strong

Both strong

HR 0.97 95% CI 0.36-2.63 log rank p=0.952

| Number of  patients at risk |  |  |  |  |  |  |
| --- | --- | --- | --- | --- | --- | --- |
| Low | 26 (0) | 23 (1) | 20 (3) | 18 (3) | 13 (7) | 9 (11) |
| High | 45 (0) | 40 (1) | 37 (1) | 33 (4) | 29 (6) | 21 (13) |


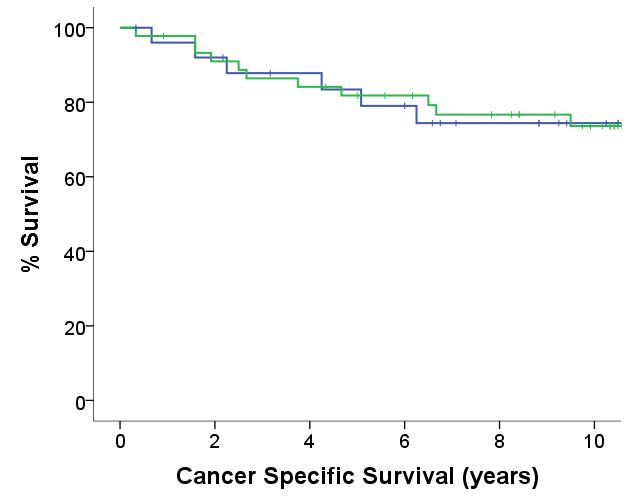


**C**

**TNM-Stage III**

Both weak or one strong

Both strong

HR 0.25 95% CI 0.10-0.64 log rank p=0.002

| Number of  patients at risk |  |  |  |  |  |  |
| --- | --- | --- | --- | --- | --- | --- |
| Low | 26 (0) | 17 (1) | 13 (1) | 8 (1) | 8 (1) | 4 (4) |
| High | 29 (0) | 24 (1) | 21 (2) | 18 (5) | 18 (5) | 15 (8) |

**
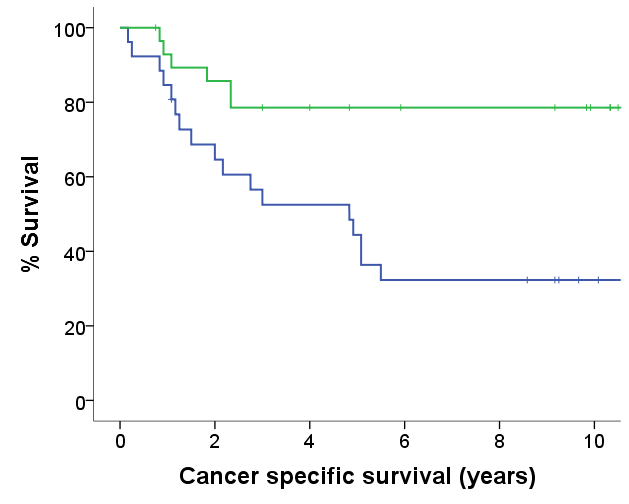
**
